# Why condition cash transfers? A multimethod pilot study evaluating the conditioning of cash transfers for tuberculosis treatment

**DOI:** 10.1186/s12913-026-15248-w

**Published:** 2026-07-30

**Authors:** Rachel Forse, Thanh Thi Nguyen, Andrew J. Codlin, Luan Nguyen Quang Vo, Lan Nguyen, Nga Nguyen, Anja M. C. Wiemers, Lan Anh Hoang, Thang Phuoc Dao, Ha Dang Thi Minh, Lan Huu Nguyen, Hoa Binh Nguyen, Dinh Van Luong, Nhung Viet Nguyen, Maxine Caws, Tom Wingfield, Knut Lönnroth, Kristi Sidney-Annerstedt

**Affiliations:** 1 Friends for International TB Relief, Hanoi, Vietnam; 2 Department of Global Public Health, Karolinska Institutet, Stockholm, Sweden; 3 IRD VN, Ho Chi Minh City, Vietnam; 4 Pham Ngoc Thach Hospital, Ho Chi Minh City, Vietnam; 5 National Lung Hospital / National TB Program, Hanoi, Vietnam; 6 University of Medicine and Pharmacy, Vietnam National University, Hanoi, Vietnam; 7 Birat Nepal Medical Trust, Kathmandu, Nepal; 8 Centre for TB Research, Liverpool School of Tropical Medicine, Department of Clinical Sciences, Liverpool, UK

**Keywords:** Tuberculosis, Cash transfers, Conditionality, Universal health coverage, Vietnam, Treatment adherence

## Abstract

**Background:**

Tuberculosis (TB) is the archetypal disease of poverty, driven by social determinants and causing catastrophic costs. A 2023 United Nations General Assembly resolution called for all TB-affected people to receive a social benefits package by 2027. Cash transfers have been shown to improve health outcomes; however, operational evidence on their implementation and effectiveness in reducing catastrophic costs due to TB is limited. This pilot study sought to assess the feasibility of delivering cash transfers to people affected by drug-susceptible TB to inform the design of a future trial.

**Methods:**

A longitudinal, non-randomized cohort study was conducted in Ho Chi Minh City, Vietnam. Half of the participants received unconditional cash transfers (UCTs) while the other half received conditional cash transfers (CCTs) after monthly appointment attendance and completion of 85% of scheduled doses. We analyzed pilot data to assess rates of participant enrollment, adherence and retention in the intervention, TB treatment success, and catastrophic cost incurrence. A 22-day time and motion study quantified the distribution of time pilot staff spent administering the cash transfer intervention by cohort.

**Results:**

Delivering cash transfers was feasible, with 60/70 (85.7%) and 60/66 (90.9%) of eligible individuals in the CCT and UCT cohorts agreeing to participate, respectively. Only 12.3% of CCT participants had one or more transfers withheld and 5.8% of the total expected CCTs were withheld. Delivering CCTs in the observation period took twice as much time as UCTs (32.4 hours for 43 CCT participants vs. 16.0 hours for 47 UCT participants). There were no significant differences in TB treatment success rates between the cohorts (91.7% CCT vs. 93.3% UCT). Cash transfers caused catastrophic cost incurrence to decline by 12.9% in the CCT cohort (44.2% to 38.5%) and 26.9% in the UCT cohort (26.8% to 19.6%).

**Conclusions:**

Cash transfers are a feasible way to mitigate the economic burden of TB in Vietnam. The conditionality assessed was not associated with any quantifiable benefits to TB-affected people, and required more effort to implement. A larger trial utilizing similar methods and outcomes is warranted. Our findings inform the design of a slightly modified approach of cash transfer delivery for the larger trial.

**Supplementary Information:**

The online version contains supplementary material available at 10.1186/s12913-026-15248-w.

## Background

Although tuberculosis (TB) has been curable with medication for over eighty years, it remains a leading infectious disease, killing approximately 1.23 million people per year [1]. There is extensive evidence that TB is both driven by socioeconomic determinants [2–5] and causes impoverishment due to income and productivity losses and the costs of accessing diagnosis and treatment [3, 6]. The Sustainable Development Goals recognize that health, poverty and wellbeing must be addressed in tandem [7–9]. Towards this end, the World Health Organization’s (WHO) End TB Strategy set a target of “zero TB-affected families facing catastrophic costs,” meaning that the costs associated with TB care do not exceed 20% of a household’s annual income and outlined multisectoral responses required to achieve this target [10].

In 2023, the United Nations General Assembly passed a resolution calling for all people with TB to have “access to a health and social benefits package so they do not have to endure financial hardship” by 2027 [11]. Social protection systems are policies and programs that aim to prevent and reduce poverty throughout the life course [12]. Broad investments in social protection have been identified as one potential method to reduce the TB burden [13], with cash transfers emerging as a promising social protection mechanism [10]. Cash transfers can be structured either as standalone, ‘TB-specific’, interventions that defray the costs of only TB care, or ‘TB-sensitive’ interventions that function as social assistance or social insurance schemes aimed to strengthen the economic resilience of households vulnerable to TB through formal social protection systems [14]. Both types of cash transfer programs have demonstrated the potential to reduce the costs of care and improve clinical outcomes, and TB-specific interventions have been shown to reduce catastrophic costs due to TB [15–17].

However, the evidence required to show effective interruption of the TB-associated medical poverty trap and mitigation of catastrophic costs from cash transfers is limited. Baseline rates of catastrophic cost incurrence due to TB have been estimated in at least 29 countries, but few interventions focusing on TB and social protection have evaluated the reduction of catastrophic costs as an outcome [18]. When TB-specific cash transfer programs have been provided at a large scale, such as in India, challenges have included delayed payments, coverage gaps, and financial support levels insufficient to reduce catastrophic costs [19–21]. Additional implementation evidence on the mechanisms by which cash transfers are delivered is required to ensure that, when provided, they achieve their intended outcomes.

Cash transfers can be delivered without conditions, known as unconditional cash transfers (UCTs) or their provision can be conditional (conditional cash transfers [CCTs]), which are based on an agreed-upon set of behaviors or actions, such as treatment adherence. Conditions can be designed to be ‘hard’ meaning that if a condition is not met, then a cash transfer is withheld, or ‘soft’ and the cash can still be delivered even if a condition goes unmet, or is met at a later date [22].

There are three primary areas of debate surrounding the conditionality of cash transfers: the ethics of mandating specific health-related behaviors in exchange for cash [23–25], the potential efficiency gains brought about by the conditions [26–30], and the *realpolitik* that there may be greater societal support for conditioning cash delivery on expected behaviors [25, 31].

The vast majority of studies evaluating whether TB-specific cash transfers improve TB clinical outcomes have utilized some form of conditionality [17]. The strongest available evidence demonstrating the benefits of cash transfers on TB outcomes comes from Brazil. The TB-sensitive Bolsa Família Program is the world’s largest CCT program and exposure to the program has been shown to significantly reduce TB incidence, mortality, and case fatality rates [16]. Qualitative evidence from Vietnam has suggested that CCTs in TB programs may have higher acceptability than UCTs [32]. UCT models that aim for universal access to cash support for all people affected by TB are potentially more in line with global targets and have also been scaled up nationally. India has implemented a TB-specific model that aims to provide small, monthly UCTs to all individuals for each month of their TB treatment [19]. In settings that are looking to establish enhanced social protection for people affected by TB through cash transfers, it is not immediately evident which delivery mechanism is most feasible, or whether the addition of conditions to a cash transfer leads to a positive effect for TB-affected people or their households.

This study aimed to evaluate the feasibility of delivering cash transfers with and without conditions to TB-affected people using multiple outcome metrics, to inform the design of a large-scale randomized controlled trial on the effectiveness of social protection to reduce catastrophic costs associated with TB in Vietnam.

## Methods

### Study design

This multimethod pilot study used a non-randomized pilot design. Non-randomized pilot studies can be used as a form of feasibility study “in which all or part of the intervention to be evaluated and other processes to be undertaken in a future trial is/are carried out (piloted)” [33]. We conducted a longitudinal cohort study to obtain data on participant enrollment rates, adherence and retention in the intervention, collected TB treatment outcomes and calculated catastrophic cost incurrence. A self-reported time and motion study was conducted to calculate the time pilot staff spent administering the intervention, by cash transfer cohort. This pilot study is reported in compliance with the CONSORT 2010 checklist [34–36].

### Study setting

**Fig. 1:**
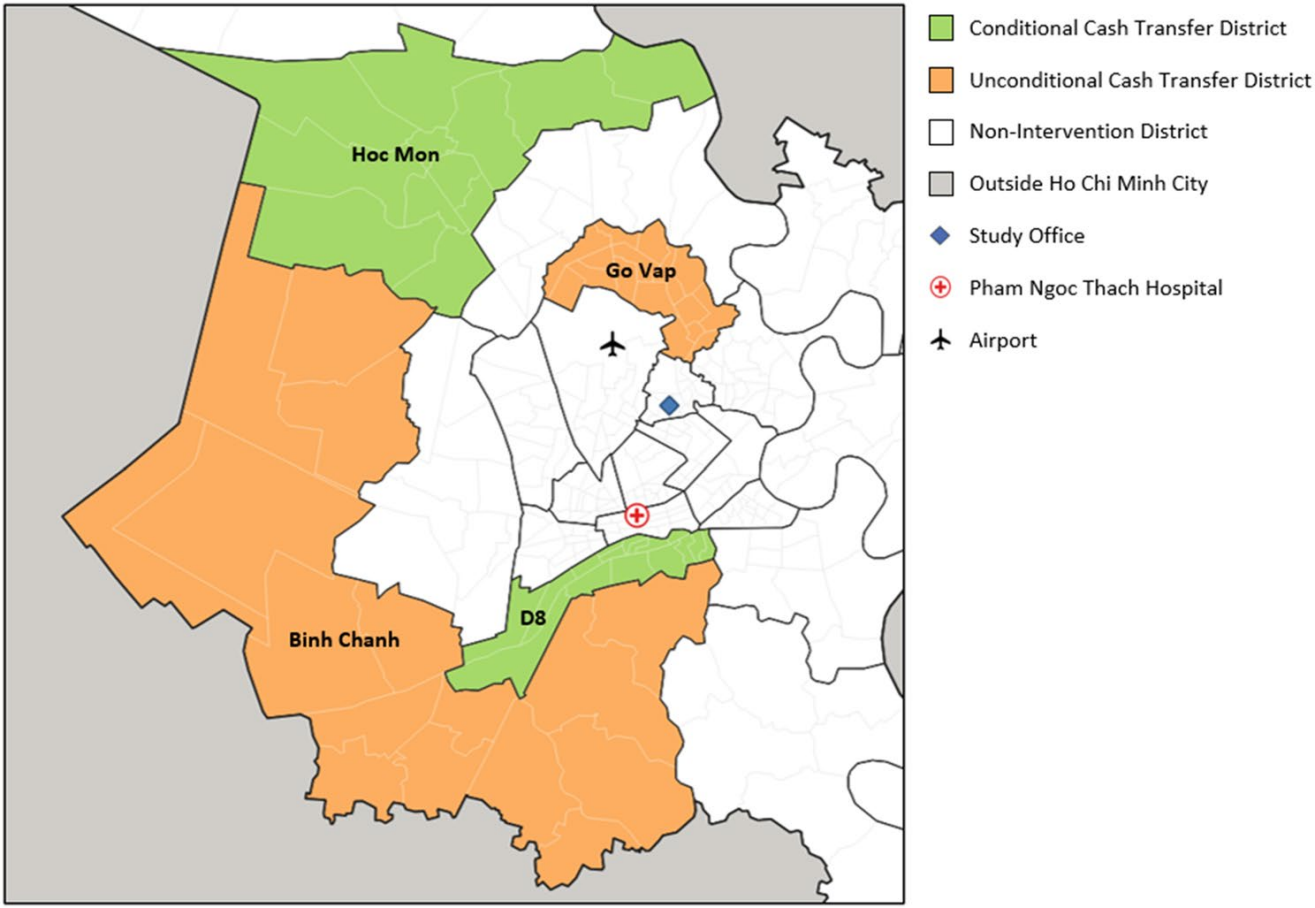
Map of the districts enrolling into the pilot study

The pilot study was conducted in four districts (District 08, Binh Chanh, Hoc Mon and Go Vap) of Ho Chi Minh City between June 2019 and July 2020 (Fig. 1). In the year prior to the study, 3,613 individuals were notified with all forms of drug-susceptible TB in the implementation districts. A convenience sample of one urban and one peri-urban district were selected for each study arm because of their high TB burden and existing collaboration on TB active case finding with Friends for International TB Relief (FIT), the implementing organization [37–39].

Training was conducted in the month before the start of the pilot for all pilot staff and District TB Unit (DTU) staff. They received separate, role-specific protocol training, with only the pilot staff trained on TB costing survey procedures, as they were solely responsible for data collection. New participant enrollment ended in December 2019, and participants were followed-up for at least six months until the end of their TB treatment. In the first months of 2020, the COVID-19 pandemic began. However, Vietnam was able to successfully contain the spread of the disease during the first 10 months of the pandemic. The pilot study experienced minimal disruptions so participants did not substantially change their TB treatment plans, pilot staff were able to freely travel to all intervention sites, and all interviews were able to be conducted in person [40, 41].

### Participants

District TB Unit (DTU) staff conducted an initial eligibility assessment for all people diagnosed within their facility to identify individuals who were diagnosed with bacteriologically-confirmed, pulmonary, drug-susceptible TB. Individuals 18 years or older, who were residing and obtaining TB treatment within an intervention district, were assessed for economic vulnerability. For an individual with TB to be included in the study, they needed to demonstrate the following indicators of vulnerability at the time of TB treatment enrollment: either not possessing social health insurance (SHI), or having a valid ‘Hộ Nghèo’ poverty status registration certificate [42]. Individuals also needed to be willing to participate and provide written consent to participate in the pilot study and associated surveys. Prior to enrollment, pilot staff verified the eligibility assessments conducted by DTU staff.

After two months of recruitment, only 11.7% and 16.7% of target enrollments had been achieved in the CCT and UCT cohorts, respectively. As a result, the pilot’s eligibility criteria were expanded to also include individuals who had been unemployed for at least 30 days prior to starting TB treatment and/or those who were employed in the informal sector and thus excluded from systems of paid sick leave and other government benefits [43].

### Intervention design

**Fig. 2:**
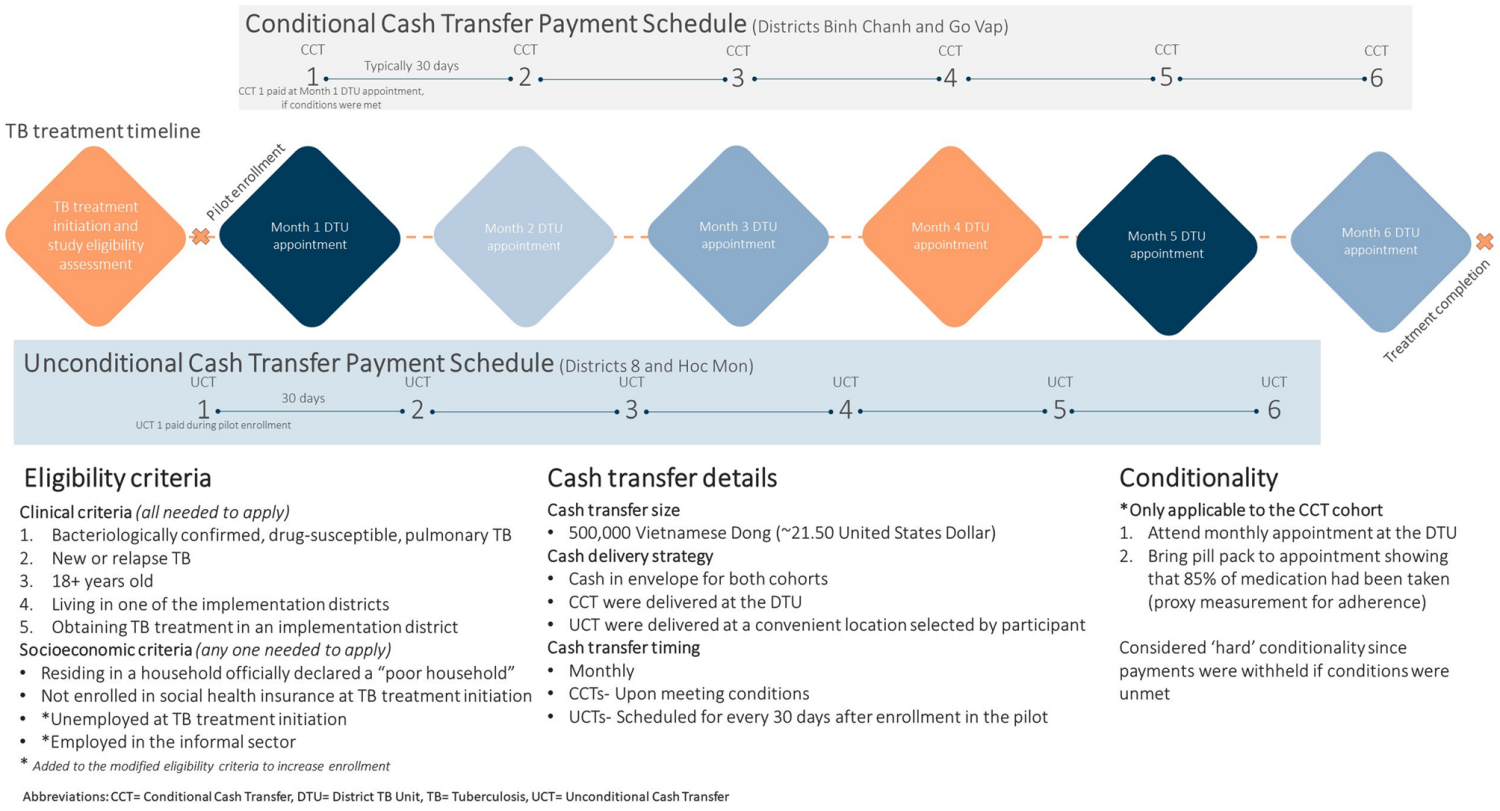
Summary of the cash transfer UHC+ intervention

The ‘cash transfer Universal Health Care (UHC)+ intervention’ provided by the pilot study included financial assistance, along with SHI enrollment for uninsured households. Figure 2 provides a visual summary of the intervention. One cohort in District 8 and Hoc Mon district received CCT, while in Binh Chanh and Go Vap districts, participants received UCT. Cash transfers of 500,000 VND (21.50 USD) were delivered monthly for up to six months. Distribution of cash had been identified as a preference over bank transfers or other forms of mobile direct benefit transfers in earlier qualitative interviews [32]. In both cohorts, the study team assisted the TB-affected households with obtaining the documentation required for SHI enrollment and subsidized the annual household SHI registration fees for individuals who did not have SHI at the start of TB treatment. These processes and their associated challenges are presented elsewhere [44]. Figure 2 presents a schematic of the intervention.

UCTs were distributed by pilot staff on a pre-arranged date at the participant’s home, the DTU, or another location selected as most convenient by the participant at study enrollment. Monthly cash transfers were delivered whether or not the participant continued in TB treatment or participated in the intervention. CCTs were distributed by DTU staff only after the beneficiary attended a monthly check-up and showed that at least 85% of the previous month’s medication had been consumed via an empty pill blister. The conditionality was considered to be ‘hard’, as those who skipped (and did not reschedule) an appointment or did not meet the proxy indicator for TB treatment adherence were not given the CCT for that month. However, the conditions were designed to be relatively easy to achieve, adding no additional costs to routine TB treatment for participants, and requiring limited documentation by DTU staff. Participants were eligible to receive the next month’s CCT if they fulfilled the conditions at their next monthly appointment, but the prior month’s transfer was not provided. The acceptability of the conditionality was assessed in conjunction with this pilot and has been reported in a separate publication [32].

### Sample size

The sample size for this pilot study was one of convenience, based on the availability of funding to support the recruitment, intervention and surveys for 120 participants, rather than the measurement of a specific endpoint related to the outcomes described above.

### Data collection

#### Intervention administration

Data were collected by pilot staff and were used to tabulate baseline characteristics of participants, monitor eligibility assessments and enrollment, adherence to the conditions among the CCT cohort, retention in the intervention, and clinical outcomes. Pilot staff continuously tracked data regarding progression through the intervention using custom-built web forms on the Organizational Network Analysis (ONA.io, NBO, Kenya) data platform. At the start of TB treatment, all participants were asked to return every 30 days for monthly follow-up appointments at the DTU. If a scheduled appointment was not attended or rescheduled within 40 days of the last appointment, then the appointment was considered ‘missed’. Upon receipt of a cash transfer in either cohort, participants signed documentation verifying the amount received. If the participant refused to sign or the value of the cash transfer was lower than 500,000 VND, an incident report form was completed to document the irregularity. Participant grievances could also be logged in these forms.

We quantified the distribution of staff time required for the various task categories, guided by the Suggested Time And Motion Procedures (STAMP) checklist [45]. Four pilot staff, including one project manager and three project officers, completed a self-reported measurement of time to the whole minute on a paper form that they carried with them throughout the day. They used the clock on their smartphone to measure time for each activity. To improve data quality, the project manager who had knowledge of the day-to-day tasks of the project officers reviewed their paper forms weekly and an independent monitoring officer reviewed the time measurements of the project manager.

Pilot staff activities were defined and coded according to three main categories and ten subcategories (Additional File 1). All task classifications were developed specifically for the study. Tasks were divided into categories for time spent directly supporting the intervention, the general administration of the intervention and research. Each task was assigned to either the CCT or UCT cohort, or it was labelled as multitasking / supporting both cohorts.

Staff time was collected after participant enrollment had been completed (24 February – 25 March 2020) to collect data on mature processes, when staff time was focused on the distribution of cash transfers and conducting surveys. Staff recorded time for their working hours (typically 9:00 to 17:00) for 22 days. Data were digitized and a monitoring officer compared the paper forms and electronic data for transcription errors.

#### TB clinical outcomes

TB treatment outcomes were exported from electronic notification registers (VITIMES) maintained by Vietnam’s National TB Program and cross-referenced with paper forms maintained at the TB treatment site [46]. Pilot staff reconciled any differences in treatment outcomes reported in VITIMES, the paper registers and patient files to obtain a final TB treatment outcome.

#### Catastrophic costs

Pilot participants were included in the catastrophic cost analysis if they had completed TB costing surveys at three time points. To calculate catastrophic costs, the WHO TB costing survey [47] was adapted into a longitudinal design (Additional File 2) [37]. Participants were asked about their direct medical and non-medical costs, time loss and indirect costs at the individual- and household-levels. First interviews collected data on all TB-related costs from first symptom onset until the day of the first interview and were conducted between two and four weeks after TB treatment initiation. Second interviews covered all TB-related costs for the period between the day after the first interview until the date of the second interview and were conducted within four weeks after the completion of the intensive phase of TB treatment. A final interview was conducted within fifteen days of the DTU recording a treatment outcome; it covered the period between the day after the second interview until the treatment outcome date.

### Statistical analyses

Descriptive statistics of enrolled participants are presented by study cohort. Binary variables were described by frequency and proportion, continuous variables by means or median according to their distribution. As applicable, Fisher’s exact, Chi-squared, or Wilcoxon rank sum tests were used to examine differences between the study cohorts. The proportion of participants receiving cash transfers was calculated for each study cohort, as well as the number and amount of individual cash transfers distributed relative to expected values. Staff time was summed by code and study cohort, and expressed as a percentage of total task time. TB treatment outcomes were described and compared across study cohorts using the Chi-squared test. The catastrophic cost calculation followed standard WHO definitions and procedures, using the human capital approach to estimate indirect costs [37, 47]. The cash transfers delivered to each participant were included as an offset against direct TB-related costs (Additional File 3), as proposed for TB-specific cash transfers by Rudgard et al. [15]. All monetary values were converted into USD (VND 1 = 0.000043 USD), based on the average conversion rate in 2022 (Exchange Rate UK). P-values below 0.05 were considered statistically significant. Data analysis was performed using Stata v17 (Stata Corp, College Station, TX, USA).

## Results

### Intervention administration

**Fig. 3:**
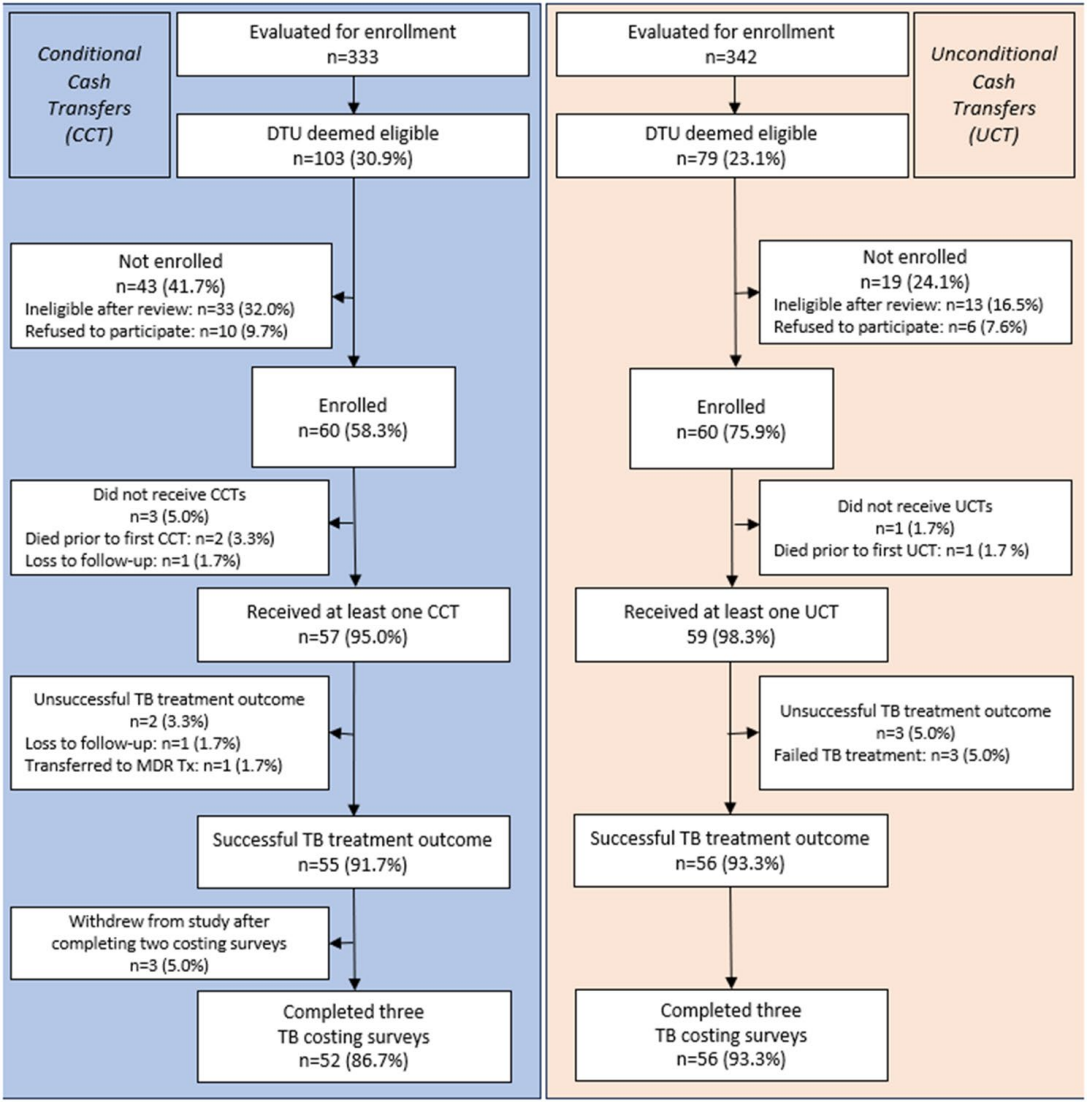
Flowchart of participant enrollment

During the recruitment period, all individuals treated for drug-susceptible TB at collaborating DTUs were assessed for eligibility in the pilot. In the CCT districts, 333 individuals were evaluated, while 342 were evaluated in the UCT districts (Fig. 3). The DTU staff deemed 103 (30.9%) eligible for enrollment in the CCT districts and 79 (23.1%) in the UCT districts. Pilot staff then reviewed and verified all of the eligibility assessments at enrollment visits; they excluded 33 (32.0%) individuals from CCT districts and 13 (16.5%) in UCT districts; the primary reason for exclusion (24/33 (72.7%) in CCT cohort; 6/13 (46.2%) in UCT cohort) was that DTU staff incorrectly indicated individuals did not possess SHI coverage, but when the pilot staff verified their eligibility data, the individuals did have a valid SHI card and therefore did not meet the economic vulnerability criteria for inclusion in the pilot. In addition, ten (9.7%) eligible individuals in the CCT districts and six (7.6%) in the UCT districts declined to participate. Participant recruitment ceased after 60 individuals were enrolled in each cohort. We attempted to complete three costing surveys for each participant; 52 (86.7%) completed all three in the CCT cohort, while 56 (93.3%) did so in the UCT cohort.

Table 1 describes the 120 enrolled participants by study cohort. Participant characteristics did not significantly differ between the cohorts in terms of gender, age, education, employment status, household size, participant’s status as head of household, health insurance coverage or poverty status. The majority of participants (70.8%) were male. Informal employment was marginally higher in the CCT cohort (60.0% CCT vs. 53.3% UCT, *p* = 0.530), while unemployment rates were the same in both cohorts at 31.7%. The median household size was four persons (IQR 3–5) in both of the study arms. SHI coverage at the start of TB treatment was marginally higher in the UCT cohort (45.0% CCT vs. 56.7% UCT, *p* = 0.201).

**Table 1:** Participant characteristics

Participant characteristics	All Participants *n* = 120	CCT Cohort *n* = 60 (Districts Binh Chanh & Go Vap)	UCT Cohort *n* = 60 (Districts 8 & Hoc Mon)	*p*-value
**Male**	85 (70.8%)	41 (68.3%)	44 (73.3%)	0.547*
**Age group**				
18–34 years	16 (13.3%)	8 (13.3%)	8 (13.3%)	0.831*
35–44 years	29 (24.2%)	15 (25.0%)	14 (23.3%)	
45–59 years	51 (42.5%)	27 (45.0%)	24 (40.0%)	
≥60 years	24 (20.0%)	10 (16.7%)	14 (23.3%)	
**Education level**				
Never attended	13 (10.8%)	5 (8.3%)	8 (13.3%)	0.619*
Primary or secondary school	85 (70.8%)	42 (70.0%)	43 (71.7%)	
High school	13 (10.8%)	7 (11.7%)	6 (10.0%)	
Vocational training, college or university	9 (7.5%)	6 (10.0%)	3 (5.0%)	
**Employment**				
Unemployed	38 (31.7%)	19 (31.7%)	19 (31.7%)	0.530*
Informally employed	68 (56.7%)	36 (60.0%)	32 (53.3%)	
Formally employed	1 (0.8%)	1 (1.7%)	0 (0.0%)	
Student	3 (2.5%)	1 (1.7%)	2 (3.3%)	
Retired	10 (8.3%)	3 (5.0%)	7 (11.7%)	
**Household size**, median (IQR)	4 (3–5)	4 (3-5.5)	4 (3–5)	0.638**
Adults, median (IQR)	3 (2–4)	3 (2–4)	3 (2–5)	0.621**
Children, median (IQR)	0 (0–1)	0 (0–1)	0 (0-1.5)	0.925**
**Participant as the head of household**	58 (48.3%)	29 (48.3%)	29 (48.3%)	1.000*
**Possessing Social Health Insurance coverage**	61 (50.8%)	27 (45.0%)	34 (56.7%)	0.201*
**Possessing ‘Hộ Nghèo’ registration**	4 (3.3%)	2 (3.3%)	2 (3.3%)	1.000*

* Chi-squared test

** Wilcoxon rank-sum (Mann–Whitney) test

Four of the 120 enrolled participants died (*n* = 3) or withdrew (*n* = 1) from the study before the first payment (Table 2). In total, 57 participants (95.0%) received cash transfers in the CCT cohort and 59 (98.3%) in the UCT cohort. In the CCT cohort, 50 participants (87.7%) received all planned cash transfers, while seven individuals (12.3%) had at least one monthly CCT withheld for inability to meet conditions. In total, 20 (5.8%) CCTs were withheld due to unmet conditions. In the UCT cohort, all 354 scheduled UCTs were paid to participants.

**Table 2:** Cash transfer distribution by study cohort

	All Participants	CCT Cohort (Districts Binh Chanh & Go Vap)	UCT Cohort (Districts 8 & Hoc Mon)
**Total number of participants enrolled the pilot project**	**120**	**60**	**60**
Died prior to cash transfer distribution	3 (2.5%)	2 (3.3%)	1 (1.7%)
Withdrew from study before any cash transfers distributed	1 (0.8%)	1 (1.7%)	0 (0%)
**Total number of participants receiving cash transfers**	**116 (96.7%)**	**57 (95.0%)**	**59 (98.3%)**
Participants receiving all planned cash transfers	109 (94.0%)	50 (87.7%)	59 (100%)
Participants missing one or more cash transfer	7 (6.0%)	7 (12.3%)	N/A
**Total number of planned cash transfers**	**696**	**342**	**354**
Cash transfers paid to participants	676 (97.1%)	322 (94.2%)	354 (100%)
Cash transfers not paid because conditions unmet	20 (2.9%)	20 (5.8%)	N/A
**Total amount of cash distributed (USD)**	**14**,**444**	**6**,**880**	**7**,**564**

Throughout the duration of the study, only one incident report form was submitted regarding the distribution of a CCT. No participants reported that the value of the cash was lower than expected or that they had been charged additional fees or forced to pay part of their cash transfer to DTU staff.

In the time and motion study, a total of 45,305 working minutes (755 hours) were self-observed and recorded by four pilot staff for the 22 days of self-observation. In this period, the CCT cohort included 43 participants receiving the intervention, while there were 47 in the UCT cohort. The distribution of total observation time (TOT) is presented in Table 3. Additional File 4 provides a detailed table with confidence intervals for all estimates. The majority of the self-observed time (83.0%) was spent on the administration of the project that was not directly related to the distribution of cash transfers, including 96.1 hours of travel time (12.7% of TOT). Direct implementation of the intervention took 91.7 hours (12.1% of TOT) and it took 47.1 hours (6.2% of TOT) to administer the cash transfer intervention in the CCT cohort vs. 39.7 hours (5.3% of TOT) in the UCT cohort. A total of five hours were spent preparing cash envelopes for participants: 1.6 hours for CCTs (0.2% of TOT) and 2.8 hours of UCTs (0.4% of TOT). Distributing cash took a total of 51.9 hours, with 32.4 hours (4.3% of TOT) spent on distribution among the CCT cohort and 16.0 hours (2.1% of TOT) in the UCT cohort. Staff spent slightly more time counselling participants in the UCT arm (13.1 hours CCT vs. 21.0 hours UCT).

**Table 3:** Distribution of time self-observed in the time and motion study

	Total Observation Time			Time for CCT Cohort (Districts Binh Chanh & Go Vap)		Time for UCT Cohort (Districts 8 & Hoc Mon)		Multitasking / supporting both cohorts	
	Hrs		%TOT	Hrs	%TOT	Hrs	%TOT	Hrs	%TOT
**Intervention**	**91.7**	**12.1%**		**47.1**	**6.2%**	**39.7**	**5.3%**	**5.0**	**0.7%**
Cash transfer preparation	5.0	0.7%		1.6	0.2%	2.8	0.4%	0.7	0.1%
Cash transfer distribution	51.9	6.9%		32.4	4.3%	16.0	2.1%	3.5	0.5%
Participant counseling	34.8	4.6%		13.1	1.7%	21.0	2.8%	0.8	0.1%
**Administration**	**627.1**	**83.0%**		**38.7**	**5.1%**	**29.1**	**3.9%**	**559.3**	**74.1%**
Travel between sites	96.1	12.7%		30.0	4.0%	23.0	3.0%	43.2	5.7%
Data management	47.3	6.3%		6.1	0.8%	5.1	0.7%	36.1	4.8%
Administrative activities	134.3	17.8%		0.7	0.1%	0.6	0.1%	133.0	17.6%
Other tasks	135.5	17.9%		1.9	0.3%	0.5	0.1%	133.2	17.6%
Team meetings	98.3	13.0%		0.0	-	0.0	-	98.3	13.0%
Breaks	115.6	15.3%		0.0	-	0.0	-	115.6	15.3%
**Research**	**36.3**	**4.8%**		**12.8**	**1.7%**	**7.8**	**1.0%**	**15.7**	**2.1%**

Hrs = Hours; TOT = Total observation time; CCT = conditional cash transfer; UCT = unconditional cash transfer

### Clinical outcomes

Table 4 presents the TB treatment outcomes by study cohort. The rate of successful treatment completion (cured or treatment completed) was 55/60 (91.7%) in the CCT cohort compared to 56/60 (93.3%) in the UCT cohort. There were no significant differences (*p* = 1.000) in treatment success rates between the study cohorts.

**Table 4:** TB treatment outcomes of participants

	Total	CCT Cohort (Districts Binh Chanh & Go Vap)	UCT Cohort (Districts 8 & Hoc Mon)	*p*-value*
**Total number of participants enrolled**	**120**	**60**	**60**	
Cured or treatment completed	111 (92.5%)	55 (91.7%)	56 (93.3%)	1.000
Failed treatment	3 (2.5%)	0 (0.0%)	3 (5.0%)	
Lost to follow up	2 (1.7%)	2 (3.3%)	0 (0.0%)	
Died	3 (2.5%)	2 (3.3%)	1 (1.7%)	
Transferred to MDR-TB treatment	1 (0.9%)	1 (1.7%)	0 (0.0%)	

* Fischer’s exact test

### Catastrophic costs

**Fig. 4:**
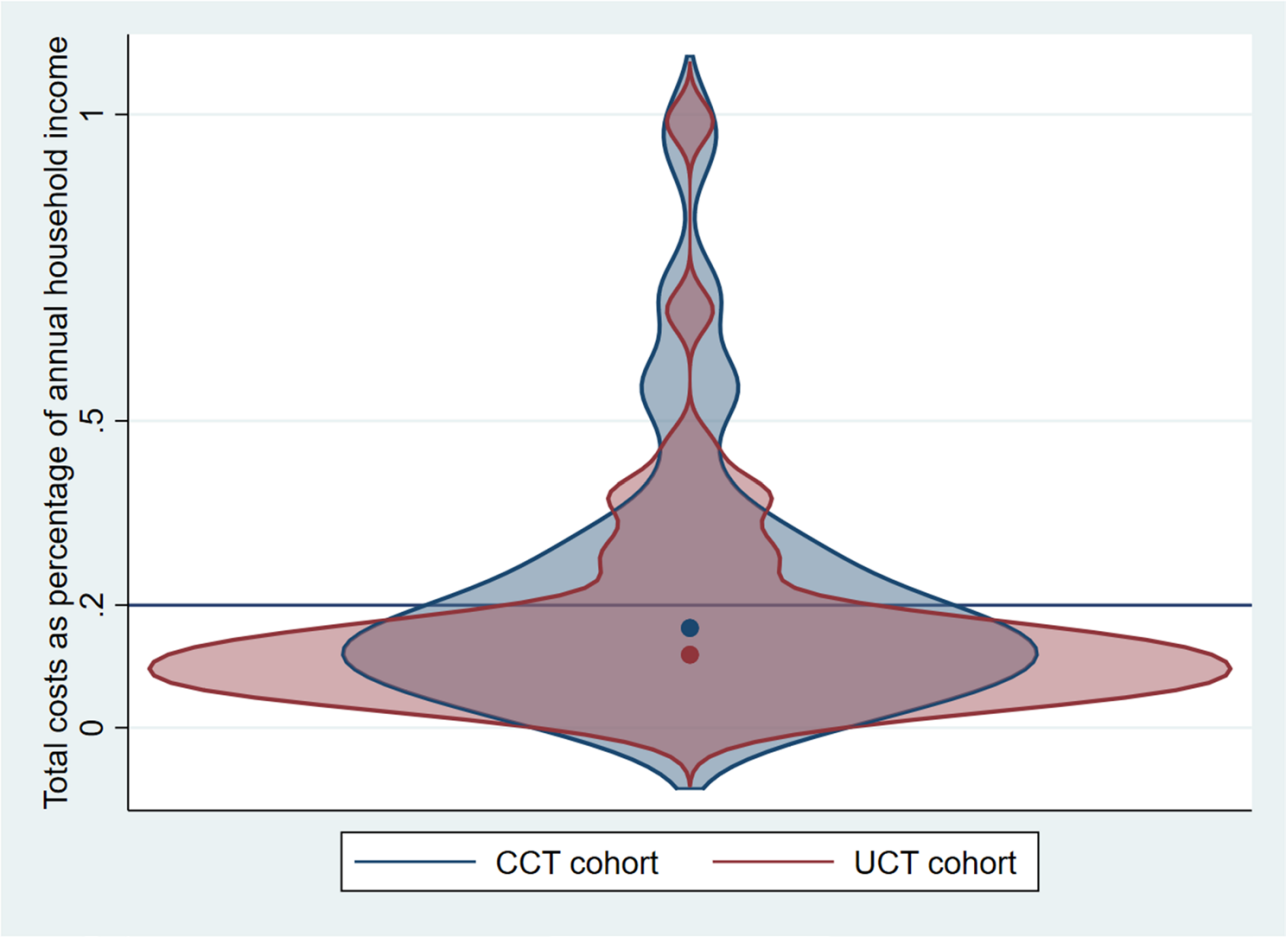
Total costs as percentage of annual household income with cash transfers included, by cohort

Catastrophic costs were calculated for the 108 participants who completed all of the three longitudinal surveys (Table 5). The total median time that TB-affected households lost was 686 hours (719 hours among CCT participants vs. 686 hours for UCT participants, *p* = 0.423). TB-affected households had total median costs of USD 1,063 (IQR 644-1,823), but there were no significant differences in total costs between the two cohorts (CCT participants lost USD 1,114 vs. UCT losses of USD 985, *p* = 0.164). For a more detailed analysis, please see Additional File 5. By including the cash transfers that were delivered in the pilot as an offset to TB costs, catastrophic cost incurrence declined by 12.9% in the CCT cohort (44.2% to 38.5%) and 26.9% in the UCT cohort (26.8% to 19.6%). There were significant differences in catastrophic cost incurrence between cohorts; 38.5% of participants (*n* = 20/52) experienced catastrophic costs in the CCT cohort compared to just 19.6% (*n* = 11/56) of participants in the UCT cohort (*p* = 0.031). The differences in catastrophic cost incurrence, at a 20% threshold, can be visualized in Fig. 4.

**Table 5:** Time loss, costs and catastrophic costs for TB-affected households

	Total (*n* = 108)	CCT cohort (*n* = 52)	UCT cohort (*n* = 56)	*p*-value
**Pre-treatment annual household income, median (IQR) in USD**	602 (387–860)	591 (333–885)	602 (398–839)	0.866*
**Median (IQR) hours lost due to TB**	686 (411–954)	719 (453-1,047)	686 (412–954)	0.423*
**Median (IQR) costs due to TB**				
Direct medical costs	152 (86–267)	163 (96–305)	143 (74–249)	0.222*
Direct non-medical costs	280 (156–452)	294 (185–498)	252 (147–414)	0.435*
Indirect costs	600 (185-1,168)	652 (265-1,308)	478 (100-1,096)	0.183*
Total costs	1,063 (644-1,823)	1,114 (699-2,015)	985 (621-1,516)	0.164*
**Proportion (IQR) of participants experiencing catastrophic costs**				
Cash transfers included	28.7 (20.9–38.0)	38.5 (26.2–52.4)	19.6 (11.1–32.3)	0.031**
Cash transfers excluded	35.2 (26.7–44.7)	44.2 (31.3–60.0)	26.8 (16.7–40.0)	0.058**

* Wilcoxon rank-sum (Mann–Whitney) test

** Fischer’s exact test

## Discussion

This study found that a pilot cash transfer UHC+ intervention using either conditional or unconditional cash transfers was able to be delivered within existing TB program services. Enrollment rates were high, with 88.2% of eligible participants successfully enrolling in the pilot. Adherence to the conditions in the CCT cohort was also high, with only 12.3% of individuals missing one or more payments and just 5.8% of total scheduled CCTs being withheld. Time spent by the program staff implementing the intervention showed that it took 6.2% of TOT to administer the CCT cohort compared to 5.3% for the UCT cohort, but the time to distribute CCTs was double that of UCTs (32.4 hours for the CCT cohort vs. 16.0 hours in the UCT cohort over 22 days of working time). TB treatment success was above 90% for both cohorts. The inclusion of cash transfers produced an 18.5% reduction in catastrophic cost incurrence among all intervention participants, with 38.5% experiencing it in the CCT cohort and 19.6% in the UCT cohort. This study has shown that it is feasible to implement both CCTs and UCTs using models of cash transfers that could be replicated in a randomized control trial or larger observational study in order to measure the effectiveness of cash transfers to alleviate catastrophic costs.

A central question of this study has been whether or not cash transfers should be conditioned on continued participation in TB treatment during a future trial. Conditionality has been critiqued for violating basic individual freedoms and by viewing participants paternalistically [25]. In addition, cash transfer interventions conditioned on treatment adherence run the risk of withdrawing cash support from individuals who need the most support and are at risk of dropping out of care [48].The pilot found no quantitative benefit of conditionality. Treatment success rates were similar between cohorts (91.7% CCT vs. 93.3% UCT), and reductions in catastrophic costs were greater in the UCT cohort despite high adherence to conditions in the CCT arm, where only 5.8% of transfers were withheld. At the same time, implementation data showed that conditionality required greater effort, particularly for cash distribution, which took twice as long in the CCT cohort. Qualitative findings from a nested acceptability study help contextualize these findings. Beneficiaries and TB healthcare providers perceived the conditions as being ethical, appropriate, and not overwhelming to implement. The conditionality was generally seen as beneficial for improving treatment adherence and fostering patient-provider relationships, but was criticized for increasing administrative burdens and causing scalability challenges [32]. Taken together, these findings indicate that while conditions may be culturally preferential and may reinforce engagement with care, they did not contribute additional measurable benefits to TB-affected people and introduced efficiency trade-offs. Therefore, future trials may consider adopting ‘soft’ conditionality, in which conditions are clearly defined to align with local norms, but do not result in withheld support [32]. Instead, failure to meet conditions should trigger additional supportive services such as enhanced counselling, psychosocial support, nutritional supplementation, transportation accommodations, and career counselling combined with job placement services, which have been identified as priority interventions by TB-affected people in Vietnam [49]. In this model, conditionality becomes a mechanism for identifying vulnerability and tailoring support, rather than restricting access to it.

To further increase equity, the targeting of financial support should be reassessed in a future study. A qualitative review of cash transfers showed the importance of properly identifying participants as it found that an inappropriate eligibility criteria can lead to negative feelings and a program to be viewed as “unfair” [24]. This pilot used a combination of clinical and socioeconomic indicators available at TB treatment initiation, utilizing a tool embedded within the TB program. Eligibility was misclassified for 25.7% of participants by DTU staff, with greater misclassification within the CCT districts, owing to the misclassification of SHI registration. During the pilot, TB treatment was provided free of charge at the DTUs and SHI could not be billed for the care or medications provided at the facility, so disclosing SHI status was not clinically relevant [44]. The high misclassification of SHI status may indicate a reticence of TB-affected people to provide government healthcare workers with accurate socioeconomic data for fear of increasing TB-treatment costs. This reticence should be considered when reassessing the eligibility criteria. Lastly, during the study, only four participants (3.3%) possessed official poverty status, entitling them to social assistance programs in Vietnam. This status is determined by comparing household income to national poverty line standards, which are lower than the cost of living in urban areas [50]. Due to its rarity in Ho Chi Minh City, this indicator is likely not an appropriate marker of eligibility within this context. A future trial should embark on a broader consultative process and develop a more appropriate risk assessment tool to target social protection support, as was completed in the Philippines [51].

This pilot used an adapted formula for calculating catastrophic costs by treating the cash transfer as an offset to direct costs [15], but retained the 20% threshold used by WHO to define catastrophic costs [47]. Catastrophic cost incurrence in the UCT cohort was significantly lower than in the CCT cohort, when the cash transfers were included. While the median costs were similar, the CCT cohort showed a broader distribution (illustrated in Fig. 4), with a larger proportion of participants incurring higher costs as a percentage of income above the 20% threshold. This suggests that a subset of individuals in the CCT cohort experienced disproportionately high costs, pushing them over the 20% catastrophic cost threshold even though the cohort-level medians did not differ significantly. The threshold-based nature of catastrophic costs (e.g., 20% of annual household income) means that even small variations in the distribution can lead to large differences in the proportion of participants exceeding the threshold. In the UCT cohort, more participants were clustered just below this threshold. The relatively small sample size could have also magnified the impact of a few outliers with higher costs in the CCT cohort, significantly influencing the proportion of participants experiencing catastrophic costs without affecting the overall median.

Currently, the WHO TB costing handbook does not provide guidance on how social assistance payments should be included in the catastrophic cost calculation [47]. The handbook should either be amended or future guidance provided to standardize these calculations across social protection interventions and increase comparability between settings.

Several additional implementation modifications should be made to the intervention before scale up. The pilot’s financial assistance delivery mode was cash. In formative qualitative work, cash had been identified as a preference [32]. However, much of the staff’s time was spent preparing cash envelopes, traveling to clinics and homes to deliver cash, and collecting documentation for verification that cash had been received. This workload could be reduced using bank transfers, e-wallet or mobile money platforms. During COVID-19, these platforms grew in popularity in Vietnam [52] and although they have been shown to create different implementation challenges (such as delays obtaining and processing correct bank details) [19], the use of digital payments should be re-evaluated with the affected population prior to the design of a scale-up intervention.

When the frequency of payment within the pilot was set at six monthly transfers, the only available data on catastrophic cost incurrence in Vietnam came from the cross-sectional national survey [53]. Subsequent longitudinal TB costing research in urban Vietnam has shown that the period with the greatest costs for TB-affected people is during the intensive phase of TB treatment [37, 54, 55]. This research indicated that it may be beneficial to provide fewer, but larger, cash transfers clustered earlier in TB treatment. While this study achieved a substantial combined 18% reduction in catastrophic cost incurrence, 28.7% of participants still experienced catastrophic costs. The size of future cash transfers should be modeled to ensure that significant reductions in catastrophic costs are achieved, and pilot schemes should explore opportunities for household income generation in addition to cash transfers.

These findings should be interpreted in light of Vietnam’s relatively strong TB program infrastructure, including routine monthly follow-up and established district-level service delivery, which may facilitate the implementation of both conditionality and cash transfer distribution. In addition, this pilot was implemented under study conditions with dedicated staff, monitoring systems, and additional resources, which may not fully reflect routine programmatic delivery. In settings with weaker health system capacity or less consistent follow-up, the feasibility and equity implications of conditionality may differ, particularly if conditions risk excluding the most underserved individuals, as has been observed in other TB cash transfer programs where implementation challenges led to delays, coverage gaps, and missed payments [19–21]. More broadly, the relative advantages of unconditional versus conditional transfers are likely to vary depending on health system capacity, social norms, and payment infrastructure.

This pilot was designed to assess the feasibility of several key components of a cash transfer program for TB in Vietnam; however, it is not without its limitations. The refusal rate among eligible individuals was 11.7%, yet comprehensive data on reasons for refusal were not collected. Future trials should systematically collect and report these data. Additionally, the grievance mechanism- the recording of incident report forms- was controlled by pilot staff and may not fully capture issues individuals faced with the intervention. A future scale-up should explore providing anonymous, digital reporting mechanisms at enrollment. Lastly, participants were not matched and a control group was not recruited so definitive claims about reductions in catastrophic costs or improvements in treatment outcomes cannot be made from this feasibility pilot. A large, randomized controlled trial is required to robustly evaluate the effectiveness of the intervention on patient outcomes.

## Conclusions

To efficiently achieve the target of “zero TB-affected families facing catastrophic costs” more evidence is needed to optimize interventions to reduce the costs of TB care. This pilot demonstrated the feasibility of a cash transfer UHC+ intervention, integrated within the National TB Program in Vietnam, and that it may be an effective strategy to substantially reduce catastrophic cost incurrence. A robust randomized controlled trial assessing reduction of catastrophic costs as a primary endpoint is warranted.

## Supplementary Information

Below is the link to the electronic supplementary material.

Supplementary Material 1

Supplementary Material 2

Supplementary Material 3

Supplementary Material 4

Supplementary Material 5

## Data Availability

The datasets used and analyzed in this study are available from the corresponding author upon reasonable request.

## Abbreviations

CCT: Conditional Cash Transfer
DTU: District TB Unit
FIT: Friends for International TB Relief
SHI: Social Health Insurance
TB: Tuberculosis
TOT: Total Observation Time
UCT: Unconditional Cash Transfer
UHC: Universal Health Care
USD: United States Dollar
VND: Vietnamese Dong
WHO: World Health Organization
