# Supplementary material for: Why condition cash transfers? A multimethod pilot study evaluating the conditioning of cash transfers for tuberculosis treatment
Source: BMC Health Serv Res. 2026 Jul 30;26:1056. doi: 10.1186/s12913-026-15248-w (PMC13439919; doi:10.1186/s12913-026-15248-w)
Supplement: Supplementary file 1 [file 12913_2026_15248_MOESM1_ESM.docx]

# **Supplementary File 1: Task code definitions**

| **Category** | **Subcategory** | **Subcategory Definition** |
| --- | --- | --- |
| Intervention | Cash transfer preparation | Time spent collecting cash from finance, bundling it, handing it over to DTU staff, collecting signatures. |
|  | Cash transfer distribution | Time spent coordinating logistics for cash distribution with participants, delivering cash to participants and/or DTUs, and collecting receipts and/or financial verification forms.  Verifying conditions- Only for CCT districts- time duration of checking pill packs and checking whether conditions had been met. |
|  | Participant counselling | Time spent (either on the phone or in-person) with participants discussing their treatment and/or the cash transfer. Time spent answering questions and fixing problems, such as calling participants about a missed appointment. |
| Administration | Travel between sites | Time duration and distance traveled on a motorbike or by foot. For this task code, record the distance travelled in kilometers (to the hundredths decimal place) on the time log. |
|  | Data management | Time spent for data entry, including examining TB patient records and registers at the District TB Units. Time spent for data entry in ONA. Time checking weekly reports, ONA, VITIMES or register data. |
|  | Administrative activities | Time spent conducting in-office financial documentation, budgeting activities, printing documents, etc. |
|  | Other tasks | Non-pilot related activities and tasks that do not fit within the definitions for the subcategories. |
|  | Team meetings | Supervisory meetings with the study implementation team. |
|  | Breaks | Breaks taken, both formal (such as a lunch break) or informal (relaxation, socializing, snacking, naps). |
| Research | TB costing surveys | Time spent collecting data, or scheduling appointments for research related to the pilot. |
