# Supplementary material for: Why condition cash transfers? A multimethod pilot study evaluating the conditioning of cash transfers for tuberculosis treatment
Source: BMC Health Serv Res. 2026 Jul 30;26:1056. doi: 10.1186/s12913-026-15248-w (PMC13439919; doi:10.1186/s12913-026-15248-w)
Supplement: Supplementary file 2 [file 12913_2026_15248_MOESM2_ESM.docx]

Interview 1 survey form:

|  | ***Part I. Participant information (to be obtained before interview)*** | | |
| --- | --- | --- | --- |
| **#** | **Question** | **Answer categories** *(circle appropriate number or fill answer on the answer line)* | ***Action for interviewer***  *The questions in part 1 are not part of the interview and should be pre-filled before the interview* |
| 1. | **Date of Interview** | ____/____/____ | (Day/month/year) |
| 2. | **Name of Province** |  |  |
| 3. | **Name of District** |  |  |
| 4. | **Place of interview (facility name)** |  |  |
| 5. | **Interviewer Name** |  |  |
| 6. | **Category of treating facility** | 1. District TB unit  2. Commune health station  3. General hospital  4. Lung hospital  5. Other____________________________ | *The "treating facility" is the place where the patient's treatment card is kept* |
| 7. | **Name of participant** |  |  |
| 8. | **Gender** | 1. Male  2. Female |  |
| 9. | **Age of participant** | ________years |  |
| 10. | **ACIS ID of participant** | ____ ____ ____ ____ ____ ____ ____ | *Fill out if available; leave blank if the person does not have an ACIS profile* |

Introduction to the patient:

***Part II. Informed consent***

My name is _______________________. The organization I am working for (Friends for International Relief and the Ho Chi Minh City Public Health Association) is interested in the costs that people face when they are treated for TB as well as the costs faced while seeking health care before the diagnosis of TB.

The information that you choose to share will be used for research purposes. It will be shared with other researchers for further analysis and published, but all your personal information will first be deleted in order to ensure full confidentiality.

It is important for you to understand that your participation in this study is completely voluntary. We would be really grateful if you would agree to participate in this study, but do feel free to decline. If you decline, there will be no consequence for you and you will receive all the care and treatment you need at the health facility as usual. If you decline to participate you will not lose any benefit that you are entitled to such as receiving care and support that is provided at the clinic.

If you decide to participate, I would like to stress that you will not receive reimbursements from the study organizers for the expenses that you report on in this interview. However, your eligibility for existing reimbursement schemes will be unaffected.

If you choose to participate in this study, you may still withdraw from the study at any stage without giving any explanation for your withdrawal. Your answers will be kept confidential. At some point I will ask you about your personal income (revenue) and the income of your household. We will NOT provide this information to any tax or welfare authorities, even after the study has been completed.

In charge of this study is the Principal Investigator <contact details>. The outcome of this study will be disseminated in an open source journal and you may request a copy from the principal investigator.

**This survey will take approximately 60-90 minutes.**

| **#** | **Question** | **Answer categories *(circle appropriate number or fill answer on the answer line)*** | | ***Action for interviewer*** |
| --- | --- | --- | --- | --- |
|  | **Do you have any questions?** | *(No response recorded)* |  | *Answer patient’s questions* |
| 11 | **Do you want to participate?** | 1. Yes  2. No | | *Yes 🡪 Thank you! Collect signature and go to interview*  *No 🡪 Ask Q11a* |
| 11a | **-> Why not?** | 1. Language not good enough  2. Time constraint  3. Not comfortable  4. Other, specify: ______________ | | *End the interview here having filled part I from patient card*  *This form should be signed by the child under 18 and guardian.* |

**Participant and/or Guardian Signature________________________________________________**

|  | **Inclusion or exclusion** | | | | | | | | | |
| --- | --- | --- | --- | --- | --- | --- | --- | --- | --- | --- |
| 12 | **Decision about inclusion or exclusion** | | | 1. Included  2. Excluded | | | | *Included🡪 skip to Q13*  *Excluded-> Ask 12b* | | |
| 12a | **->If excluded, reason for exclusion** | | | 1. No informed consent  2. Treatment registration group is “other” | | | | *After completing this question, the survey is completed and this patient excluded from the survey.* | | |
| 13 | **Interviewee identity** | | | 1. Patient  2. Guardian  3. Other (please name) ____________________ | | | |  | | |
|  | | | | | | | | | |  |
| ***Part III- Costs before the current TB treatment (filled for new cases in intensive phase only)*** | | | | | | | | | |  |
|  | | | | | | | | | |  |
|  | | **O*ut-of-pocket expenditure, reimbursements and time loss before and during TB diagnosis (before start of TB treatment)*** | | | | | | | |  |
| 14 | | **Do you currently have any health insurance?** | 1. Yes  2. No | | | | | | *Yes 🡪 Ask Q14a*  *No 🡪 Skip to Q15* |  |
| 14a | | **-> What type of health insurance do you have?** | 1. Mandatory- company purchases for employees  2. Voluntary- Paid out of pocket (typically for workers in the informal sector)  3. Private  4. Other:_____________________ | | | | | | *Ask if Q14 is 1. Yes* |  |
| 14b | | **-> What is the health insurance number?** | ______ | | | | | | *Ask if Q14 is 1. Yes*  *Review health insurance card and collect type of insurance. Limit to 1-5.* |  |
| 14c | | **-> Select health insurance 2 letter code:** | BT; CA; CB; CC; CH; CK; CN; CS; CT; CY; DK | | DN; DT; GB; GD; HC; HD; HG; HN; HS; HT; HX | KC; LS; MS; NN; NO; PV; QN; SV; TA; TB; TC | TE; TK; TN; TQ; TS; TY; XB; XD; XK; XN | | *Ask if Q14 is 1. Yes*  *Select one based on the insurance card. See interviewer guidelines at the end for more details.* |  |
| 14d | | **-> What percentage of health expenditure does your insurance cover?** | ____% | | | | | | *Ask if Q14 is 1. Yes*  *See interviewer guidelines at the end for more details.* |  |
| 143. | | **-> At which hospital are you registered?** | ____________________________________ | | | | | | *Ask if Q14 is 1. Yes* |  |
| 15 | | **Did you experience symptoms of TB before you were diagnosed with this TB episode?** | 1. Yes  2. No | | | | | | *First construct a timeline of events, either starting with the first TB symptom, or start with time of TB diagnosis and work backwards. To help the patient remember when the illness started, you can ask which TB symptom was first experienced.*  *Probe for* ***cough, weight loss, chest pain, night sweats****.*  *If there is a problem defining the difference between TB symptoms and other health problems, ask which symptom led the patient to seek care, then ask when that symptom first occurred or became worse and started to worry the patient.*  *If 2.No, skip to Q16* |  |
| 15a | | **-> When did you first experience symptoms of TB for this TB episode?** | Number of weeks before treatment started: ________ | | | | | |  |  |

| 16 | **Before your TB treatment started, did you seek care or advice for your symptoms of the current illness at either hospitals, commune health stations, district TB units, district health centres, pharmacies, or a private doctor?** | 1. Yes  2. No | *Yes 🡪 Fill out table for Q16a and Q16b*  *No 🡪 Skip to Q18 on page 7* |
| --- | --- | --- | --- |
|  | **-> We would like to construct a timeline for every visit that you made to different providers for the symptoms of your current illness*.*** *(Enter in chronological order in 16a and 16b)* | **16a. Provider type**   1. Commune Health Station 2. District TB Unit 3. District Health Centre 4. Pharmacy/Drugstore 5. Private clinic 6. Public hospital where the patient is registered 7. Public hospital- **not** where the patient is registered 8. Private hospital 9. Herbalist/traditional practitioners 10. Other__________________________ | **16b. How many weeks before starting TB treatment in the current facility did you visit each of these providers?** |
|  | **1^st^ visit** | Provider category: ________ | Number of weeks before treatment started: ________ |
|  | **2^nd^ visit** | Provider category: ________ | Number of weeks before treatment started: ________ |
|  | **3^rd^ visit** | Provider category: ________ | Number of weeks before treatment started: ________ |
|  | **4^th^ visit** | Provider category: ________ | Number of weeks before treatment started: ________ |
|  | **5^th^ visit** | Provider category: ________ | Number of weeks before treatment started: ________ |
|  | **6^th^ visit** | Provider category: ________ | Number of weeks before treatment started: ________ |
|  | **7^th^ visit** | Provider category: ________ | Number of weeks before treatment started: ________ |
|  | **8^th^ visit** | Provider category: ________ | Number of weeks before treatment started: ________ |
|  | **9^th^ visit** | Provider category: ________ | Number of weeks before treatment started: ________ |
|  | **10^th^ visit** | Provider category: ________ | Number of weeks before treatment started: ________ |

**17. How much money and time did you spend for each of these visits before you were diagnosed with TB, including the visit when you actually received your diagnosis? (For patient, guardian and the person accompanying the Patient)**

|  | | | |  | Medical out-of-pocket payments,  (Total per visit)  (A) | | | | | | | | Non-medical out-of-pocket payments,  (Total per visit)  (B) | | | | | Health insurance contribution | | |
| --- | --- | --- | --- | --- | --- | --- | --- | --- | --- | --- | --- | --- | --- | --- | --- | --- | --- | --- | --- | --- |
| Visit  *(corresponds with table for Q16)* | Provider Type  (corresponds to Q16) | # of days hospitalized (for hospitalizations only) | Travel time and time spent for visit (Hours) | Daily room charges  (for hospitalizations only)  A1 | Consultation fee  (total for stay)  A2 | Radiography and other imaging  A3 | Lab  tests  A4 | Other procedure  A5 | Medicines  (do not include TB medicines)  A6 | TB medicines (do not include free meds)  A7 | Other medicines, incl nutritional supple-ments  A8 | Un-itemized medical payments  A9 | Travel  B1 | Food during health care visit or hospital stay  B2 | Other, including accommo-dation  B3 | Un-itemized Non-medical out-of-pocket payments  B4 | On this visit did you use health insurance? | | Health insurance reimbursement to patient  (C) |  |
| 1st |  |  |  |  |  |  |  |  |  |  |  |  |  |  |  |  | Y/N | |  |  |
| 2nd |  |  |  |  |  |  |  |  |  |  |  |  |  |  |  |  | Y/N | |  |  |
| 3rd |  |  |  |  |  |  |  |  |  |  |  |  |  |  |  |  | Y/N | |  |  |
| 4th |  |  |  |  |  |  |  |  |  |  |  |  |  |  |  |  | Y/N | |  |  |
| 5th |  |  |  |  |  |  |  |  |  |  |  |  |  |  |  |  | Y/N | |  |  |
| 6th |  |  |  |  |  |  |  |  |  |  |  |  |  |  |  |  | Y/N | |  |  |
| 7th |  |  |  |  |  |  |  |  |  |  |  |  |  |  |  |  | Y/N | |  |  |
| 8th |  |  |  |  |  |  |  |  |  |  |  |  |  |  |  |  | Y/N | |  |  |
| 9th |  |  |  |  |  |  |  |  |  |  |  |  |  |  |  |  | Y/N | |  |  |
| 10th |  |  |  |  |  |  |  |  |  |  |  |  |  |  |  |  | Y/N | |  |  |

|  | ***Part IV. Cost during current TB/MDR-TB treatment (to be filled for all participants)***  ***This section refers to the patient’s current treatment phase only*** | | |
| --- | --- | --- | --- |
| 18 | **After starting your TB treatment until now, did you seek care or advice for your symptoms of the current illness at either hospitals, commune health stations, District TB Units, District Health Centres, pharmacies, or a private doctor?** | 1. Yes  2. No | *If yes, answer 18a and 18b*  *If no, skip to Q19.* |
|  | **-> Let’s construct a timeline for every visit that you made to different providers for the symptoms of your current illness, since you began TB treatment*.*** *(Enter in chronological order in 18a and 18b)* | **18a. Provider type**   11. Commune Health Station 12. District TB Unit 13. District Health Centre 14. Pharmacy/Drugstore 15. Private clinic 16. Public hospital 17. Private hospital 18. Herbalist/traditional practitioners 19. Other | **18b. Number of weeks** |
|  | **1^st^ visit** | Provider category: ________ | Number of weeks since treatment started: ________ |
|  | **2^nd^ visit** | Provider category: ________ | Number of weeks since treatment started: ________ |
|  | **3^rd^ visit** | Provider category: ________ | Number of weeks since treatment started: ________ |
|  | **4^th^ visit** | Provider category: ________ | Number of weeks since treatment started: ________ |
|  | **5^th^ visit** | Provider category: ________ | Number of weeks since treatment started: ________ |
|  | **6^th^ visit** | Provider category: ________ | Number of weeks since treatment started: ________ |
|  | **7^th^ visit** | Provider category: ________ | Number of weeks since treatment started: ________ |
|  | **8^th^ visit** | Provider category: ________ | Number of weeks since treatment started: ________ |
|  | **9^th^ visit** | Provider category: ________ | Number of weeks since treatment started: ________ |
|  | **10^th^ visit** | Provider category: ________ | Number of weeks since treatment started: ________ |

| 19. | **Are you currently hospitalized?** | 1. Yes  2. No | *If yes, the cost data collected applies to the first row of the table in Q22* |
| --- | --- | --- | --- |
| 20 | **Have you been previously hospitalized or sought emergency care since you started TB treatment and because of TB?** | 1. Yes  2. No | *Concerns only hospitalization during the current treatment phase: Does not include hospitalization before the current TB treatment started:*  *For new cases, hospitalizations prior to TB treatment started should be filled in part III.* |
| 20a | **-> If yes, how many times?** | _______ times |  |
| 21 | **Did somebody in your household accompany you for your last hospitalization?** | 1. Yes  2. No | *If there were no hospitalizations in Q18, Q19 or Q20, then skip to Q23 on page10.* |

22. **About how much money and time did you spend for each of these hospitalizations/emergency care? (For patient, guardian and/or the person accompanying the patient)**

|  | | | |  | Medical out-of-pocket payments,  (Total per visit)  (A) | | | | | | | | Non-medical out-of-pocket payments,  (Total per visit)  (B) | | | | Health insurance contribution | |
| --- | --- | --- | --- | --- | --- | --- | --- | --- | --- | --- | --- | --- | --- | --- | --- | --- | --- | --- |
| Visit  *(corresponds with table for Q18)* | Provider Type  (corresponds to Q18) | # of days hospitalized (for hospitalizations only) | Travel time and time spent for visit (Hours) | Daily room charges  (for hospitalizations only)  A1 | Consultation fee  (total for stay)  A2 | Radiography and other imaging  A3 | Lab  tests  A4 | Other procedure  A5 | Medicines  (do not include TB medicines)  A6 | TB medicines (do not include free meds)  A7 | Other medicines, incl nutritional supplements  A8 | Un-itemized medical payments  A9 | Travel  B1 | Food during health care visit or hospital stay  B2 | Other, including accommo-  dation  B3 | Un-itemized Non-medical out-of-pocket payments  B4 | On this visit did you use health insurance? | Health insurance reimbursement to patient  (C) |
| 1st |  |  |  |  |  |  |  |  |  |  |  |  |  |  |  |  | Y/N |  |
| 2nd |  |  |  |  |  |  |  |  |  |  |  |  |  |  |  |  | Y/N |  |
| 3rd |  |  |  |  |  |  |  |  |  |  |  |  |  |  |  |  | Y/N |  |
| 4th |  |  |  |  |  |  |  |  |  |  |  |  |  |  |  |  | Y/N |  |
| 5th |  |  |  |  |  |  |  |  |  |  |  |  |  |  |  |  | Y/N |  |
| 6th |  |  |  |  |  |  |  |  |  |  |  |  |  |  |  |  | Y/N |  |
| 7th |  |  |  |  |  |  |  |  |  |  |  |  |  |  |  |  | Y/N |  |
| 8th |  |  |  |  |  |  |  |  |  |  |  |  |  |  |  |  | Y/N |  |
| 9th |  |  |  |  |  |  |  |  |  |  |  |  |  |  |  |  | Y/N |  |
| 10th |  |  |  |  |  |  |  |  |  |  |  |  |  |  |  |  | Y/N |  |

|  | ***Costs for DOT and food costs during ambulatory care***  ***(****DOT (Directly observed treatment) visit is for the supervision of daily intake of medicines, i.e, what is done every day. These questions are not referring to less frequent trips to pick up drugs (e.g., weekly), which are explored from Q24 onwards.)* | | | |
| --- | --- | --- | --- | --- |
| 23. | **On a daily basis, do you currently take your medicines yourself without supervision or support (self-administered) or do you have a treatment supervisor or supporter (DOT)?** | 1. Self-administered  2. DOT via DTU  3. DOT via DTU/commune health station | - *If 1. self-administered skip to Q24* - *If 2. DOT via DTU or 3. DTU/commune health station, ask Q23a- Q23g* |  |
| 23a. | **-> For any form of DOT, how many times a week?** | _______ Times/week | *The maximum will be 7 times a week* |  |
| 23b. | **-> Who is your DOT provider/supporter?** | 1. Commune health station  2. DTU officer  3. Community health workers (CTV) or TB counsellors (TVV)  4. Work place  5. Family member  6. Other_______________ |  |  |
| 23c. | **-> How long did the last DOT visit take, including travel time and waiting time (total turnaround time)?** | ________Minutes |  |  |
| 23d. | **-> What was the cost of transport (return) for the last DOT visit, including parking costs, in total for you and any accompanying household member?** | ________ VND |  |  |
| 23e. | **-> How much did you spend on food, drinks and accommodation for the last DOT visit (on the road, while waiting, lunch etc.), in total for you and any accompanying household member?** | ________ VND |  |  |
| 23f. | **-> If there were any other fees or costs, how much did you spend on other fees during your last DOT visit?** | ________ VND | *Include: plastic bags for sputum transport, cost of injections, travel for a DTU officer to the patient’s home, etc..*  *If no other costs, then answer 0 VND* |  |
| 23g. | **-> Did somebody in your household accompany you for your last DOT visit?** | 1. Yes  2. No |  |  |

|  | ***Costs of picking up drugs and food costs during ambulatory care***  *This section does not concern DOT visits, which should have recorded in the last section, but should filled if patient or other household member picks up drugs for either bringing to DOT provider or for self-administered treatment.* | | | |
| --- | --- | --- | --- | --- |
| 24. | **Do you or a household member pick up TB drugs (for self-administered treatment or to bring to your DOT supervisor/supporter)?** | 1. Yes  2. No | *If patient is on DOT and patient or household member is* ***not*** *picking up drugs to bring to DOT provider, then the answer is no.*  *If yes, then answer 24a.-24g.*  *If no, skip to Q25 on page 12* |  |
| 24a. | **-> How often do you or a household member pick up TB drugs in the current treatment phase?** | 1. Every day  2. Every week  3. Every 2 weeks  4. Every month  5. Other____________ |  |  |
| 24b. | **->Where do you or your household member pick up your TB drugs?** | 1. Commune health station  2. District TB unit  3. Other:________ | *If the patient has visited different places, tick the most recent one.* |  |
| 24c. | **-> What accommodation cost did you and any accompanying household member have when you last picked up drugs?** | ________ VND |  |  |
| 24d. | **-> How long did the last visit to pick up drugs take, including travel time and waiting time (total turnaround time)?** | ________Minutes |  |  |
| 24e. | **-> What was the cost of transport (return) last time you picked up drugs, including parking costs, in total for you and any accompanying household member?** | ________ VND |  |  |
| 24f. | **-> How much did you spend on food and drinks last time you picked up drugs (on the road, while waiting, lunch etc.), in total for you and any accompanying household member?** | ________ VND |  |  |
| 24g. | **-> If there were any other fees or costs, how much did you spend on these other fees the last time you picked up drugs, in total, for you and any accompanying household member?** | ________ VND | *If no other costs, then answer 0 VND* |  |
| 24h. | **-> Did somebody in your household accompany you for your last visit to pick up drugs or did they pick up drugs for you?** | 1. Yes  2. No |  |  |

|  | ***Cost during outpatient visits for medical follow-up (see the doctor or nurse, have tests)*** | | |
| --- | --- | --- | --- |
| 25. | **How many TB-related medical follow-up visits have you had so far (to see the doctor or nurse, have follow- up tests, etc.)?** | ________ Times | *This concerns clinical check-up, follow up, and additional visits due to side effects or other TB related issues. It does not include DOT visits or visits to pick up drugs.* |
| 26. | **How long did the last follow-up medical outpatient visit take, including travel time and waiting time (total turnaround time)?** | ________ Hours |  |
| 27. | **What was the cost of transport (return) at the last follow-up medical outpatient visit, including parking, in total for you and any accompanying household member?** | ________ VND | *Cost related to the latest visit. If the interview takes place at the end of such a visit use the costs for the present visit for Q27-36* |
| 28. | **How much did you spend on food and drinks at the last follow-up medical outpatient visit (on the road, while waiting, lunch etc.), in total for you and any accompanying household member?** | ________ VND |  |
| 29. | **What accommodation cost did you have for the last follow-up medical outpatient visit, in total, for you and any accompanying household member?** | ________ VND |  |
| 30. | **What fees did you pay during your last follow-up medical outpatient visit for registration/consultation?** | ________ VND |  |
| 31. | **What fees did you pay during your last follow-up medical outpatient visit for radiography and other imaging?** | ________ VND |  |
| 32. | **What fees did you pay during your last follow-up medical outpatient visit for tests, TB tests and others?** | ________ VND |  |
| 33. | **What fees did you pay during your last follow-up medical outpatient visit for other procedures?** | ________ VND |  |
| 34. | **What fees did you pay at your last follow-up medical outpatient visit for TB medicines, including prescriptions for medicines bought outside the facility?** | ________ VND |  |
| 35. | **What fees did you pay during your last follow-up medical outpatient visit for other medicines, including nutritional supplements?** | ________ VND |  |
| 36. | **What other fees not listed in the previous questions did you pay during your last follow-up medical outpatient visit?** | ________ VND | *Examples include: masks, health staff fee (unofficial)* |
| 37. | **Did somebody in your household accompany you for your last medical follow-up visit?** | 1. Yes  2. No |  |

|  | ***Costs for nutritional/food supplements*** | | | |  |
| --- | --- | --- | --- | --- | --- |
| 38. | **Do you buy any nutritional supplements outside your regular diet because of the TB illness, for example vitamins, meat, energy drinks, or fruits as recommended by health care staff?** | 1. Yes  2. No | | *If yes, ask Q38a*  *If no, skip to Q39* | |
| 38a. | **-> How much did you spend on nutritional supplements (vitamins, meat, energy drinks, or fruits) in the past week approximately?** | ________ VND | |  | |
|  | ***Time loss for guardians***   - ***Not to be filled if the patient is under 15 years – for children, all questions concerning costs, time spent, income, and income loss in sections III and IV concern cost for the guardian.*** - ***Note: out-of-pocket costs of transport, food, accommodation for guardian should be included in questions on Part IV (tables).*** | | | |  |
| 39. | Earlier in the survey, you indicated that others have accompanied you as you sought care for TB and we collected the out-of-pocket costs for the person who accompanied you**. Can you identify the last time someone accompanied you as you sought healthcare and the type of visit that you were going for?** | 0. No one has accompanied me  1. Hospitalization  2. DOT visit  3. Drug pick-up  4. Medical follow-up visit | *If yes, to Q21, Q23g, Q24h or Q37, then responses to Q39 should be 1-4.*  *If Q39 is 0. No one accompanied me, then skip to Q40* | |  |
| 39a. | **->Who accompanied you to that visit?** | 1. Grandfather  2. Grandmother  3. Father  4. Mother  5. Uncle  6. Aunt  7. Husband  8. Wife  9. Brother  10. Sister  11. Cousin (male)  12. Cousin (female)  13. Child (son)  14. Child (daughter)  15. Friend  16. Other household member________ |  | |  |
| 39b. | **->During that last visit, did the person who accompanied you, take time off work?** | 1. Yes  2. No |  | |  |
| 39c. | **->Did that person lose any income as they accompanied you for the visit?** | 1. Yes  2. No |  | |  |
| 39d. | **->How much income would you estimate that the person who accompanied you lost during your last visit?** | ________ VND | *If Q39c is 1. Yes, then ask 39d.*  *If Q39c is 2. No, then skip to Q40* | |  |

|  | ***PART V:***  ***Social position*** | | |
| --- | --- | --- | --- |
| 40 | **How many people are in your household?** | ______ people | *The interviewer should work with the interviewee to list out the names of the people who live under the same roof as the patient, share a kitchen and have been living there for at least one month. See template in interviewer guidelines.* |
| 40a | **-> Number of adults** | ________ Adults |  |
| 40b | **-> Number of children** | ________ Children |  |
| 41. | **Are you the head of your household?** | 1. Yes  2. No | *The HoH is the person listed in the family book as the HoH -or- the person that family members trust and respect most.* |
| 41a. | **-> Who is the head of your household?** | 1. Grandfather  2. Grandmother  3. Father  4. Mother  5. Uncle  6. Aunt  7. Husband  8. Wife  9. Brother  10. Sister  11. Cousin (male)  12. Cousin (female)  13. Child (son)  14. Child (daughter)  15. Friend  16. Other household member________ | *Ask if 41 is 2.No*  *Skip if 41 is 1.Yes* |
| 41b. | **->What education level did the head of the household complete?** | *1. Not literate*  *2. Literate*  *3. Primary school*  *4. Secondary school*  *5. High school*  *6. Technical school*  *7. College/University*  *8. Post-graduate* | *Ask if 41 is 2.No*  *Skip if 41 is 1.Yes* |
| 41c. | **->How many total years of education did the head of household complete?** | *________ Years* | *Ask if 41 is 2.No*  *Skip if 41 is 1.Yes* |
| 42. | **What education level did you complete?** | *1. Not literate*  *2. Literate*  *3. Primary school*  *4. Secondary school*  *5. High school*  *6. Technical school*  *7. College/University*  *8. Post-graduate* | *If patient is under 15 years, this question is for the guardian.* |
| 43. | **How many total years of education did you complete?** | *________ Years* |  |
| 44. | What is your main occupation? | 1. School student 2. Technician 3. Service 4. Factory worker 5. Farmer 6. Government employee 7. Teacher 8. Retiree 9. Homemaker 10. Unemployed 11. Manual labor 12. Microenterprise owner 13. Other (specify): ________ | *If patient is under 15 years, this question is for the guardian.*  *This section will be adapted to the local occupation definitions (ref. Statistical dpt/Household survey categories)* |
| 45. | What was your primary employment, or normal work, or normal other main activity before you contracted TB? | 0. Unemployed  1. Formal paid work  2. Informal paid work  3. Retired  4. Student  5. Housework  6. Other (specify): ________ | *If patient is under 15 years, this question is for the guardian.*  *This refers to the time before TB symptoms developed. Name all options first*  *“3.Retired” is defined as when a person is no longer working because of their age. It does not only refer to government employees who receive a pension.* |
| 46. | What is your primary employment, or normal work, or normal other main activity now? | 0. Unemployed  1. Formal paid work  2. Informal paid work  3. Retired  4. Student  5. Housework  6. Other (specify): ________ | *If patient is under 15 years, this question is for the guardian.*  *This refers to the time after TB symptoms developed. Name all options first* |

|  | **Constructing a socio-economic status index with household asset questions.** | | |
| --- | --- | --- | --- |
| 47. | **What is your usual main source of drinking/ cooking water?** | 1. Piped or Bottled 2. Well 3. Other | *Other includes all sources that are not from a piped source, bottle, or well. This includes natural spring, borehole, rainwater, etc.* |
| 48. | **What kind of toilet facilities do you have?** | 1. Flush toilet 2. Other |  |
| 49. | **Does your household have?** |  |  |

|  | Electricity | 1. Yes 2. No |  |
| --- | --- | --- | --- |
|  | Mobile Telephone | 1. Yes 2. No |  |
|  | Fixed Telephone | 1. Yes 2. No |  |
|  | Refrigerator | 1. Yes 2. No |  |
|  | Computer | 1. Yes 2. No |  |
|  | Fan | 1. Yes 2. No |  |
|  | Watch | 1. Yes 2. No |  |
|  | Bicycle | 1. Yes 2. No |  |
|  | Television | 1. Yes 2. No |  |
|  | Manual scooter | 1. Yes 2. No |  |
|  | Automatic scooter | 1. Yes 2. No |  |
|  | Car | 1. Yes 2. No |  |
|  | Sewing machine | 1. Yes 2. No |  |
|  | Stereo equipment | 1. Yes 2. No |  |
|  | Air conditioner | 1. Yes 2. No |  |
|  | Washing machine | 1. Yes 2. No |  |
|  | Drying machine (dryer) | 1. Yes 2. No |  |
|  | Water heater | 1. Yes 2. No |  |
|  | Gas stove | 1. Yes 2. No |  |
|  | Induction cooker | 1. Yes 2. No |  |
|  | Rice cooker | 1. Yes 2. No |  |
|  | Vacuum cleaner | 1. Yes 2. No |  |
|  | Dehumidifier | 1. Yes 2. No |  |
|  | Water filter | 1. Yes 2. No |  |
|  | Microwave | 1. Yes 2. No |  |
|  | Oven | 1. Yes 2. No |  |
|  | Fax machine | 1. Yes 2. No |  |
|  | Wheelchair | 1. Yes 2. No |  |

|  | ***Income (reported) before contracting TB (****This refers to the time before TB symptoms developed.)* | | | | |
| --- | --- | --- | --- | --- | --- |
| 50. | Were you the person who earned the highest income in your household before you contracted TB? | | 1. Yes  2. No | *For this section, if patient is under 15 years, this question is for the guardian.* | |
| 51. | How many hours a week were you working before you contracted TB? | | ________ hours/week |  | |
| 52. | If you were in paid work, how much do you estimate your take-home (net) income from labour related activities, per month was before you contracted TB? | | ________ VND | *If patient is under 15 years, this question is for the guardian. If the patient reports 0 net income, then the interviewer should record 0 in Q52. and at the end of the survey, in Q65, details about how the person managed to live without income should be recorded.* | |
| 53. | How much was your estimated income each month from other sources (not labor-related) before you contracted TB? | | ________ VND | *Include income from owning rentals, interest from investments, etc… If no other income, mark 0 VND.* | |
| 54. | We are interested in understanding the income of your household before you contracted TB. We previously listed all of the people in your household. Can you estimate how much income each person in your household earned (take-home/net income) per month from labour related activities before you contracted TB?  *Enter:* Estimated net income from labour related activities of the household was per month, before you contracted TB? | | ________ VND | *The interviewer should go through the list of people created in Q40. and ask if each person was working before the patient contracted TB. The interviewer should write down the estimated income from labour related activities for each person. Once the interviewer has obtained an estimated income for each household member, the interviewer should add the estimated income for each person in the household and enter the total here.* | |
| 55. | Can you estimate how much income each person in your household earned (take-home/net income) per month from non-labour related activities before you contracted TB? | | ________ VND | *Include income from owning rentals, interest from investments, etc… If no other income, mark 0 VND.* | |
|  | ***Income changes and social consequences*** | | | | |
| 56. | **Are you currently working?** | 1. Yes  2. No | | | *For this section, if patient is under 15 years, this question is for the guardian.* |
| 56a | **-> If yes, how many hours per week are you working now?** | ________ hours/week | | | *Ask if Q56 is 1.Yes* |
| 56b. | **-> If yes, what is your current monthly take-home income (net)?** | ________ VND | | | *Ask if Q56 is 1.Yes* |
| 56c. | **-> If yes, how many working days have you lost because of TB?** | ________ Days | | | *Ask if Q56 is 1.Yes*  *Working days: e.g., if a patient was not able to work for 5 half days and lost income for these, the number of days lost is 0.5*5=2.5.* |
| 56d. | **-> If no, when did you stop working?** | ____/____/____ | | | *Ask if Q56 is 2. No*  *Day/month/year* |
| 56e. | **-> If no, why did you stop working?** | 1.Illness related to TB  2. Not related to TB | | | *Ask if Q56 is 2. No* |
| 56f. | **-> If no, how many working days did you lose before you stopped working entirely?** | ________ Days | | | *Ask if Q56 is 2. No*  *Working days: e.g., if a patient was not able to work for 5 half days and lost income for these, the number of days lost is 0.5*5=2.5. Report for all days before job loss.* |
| 57. | We are interested in understanding the current income of your household. We previously listed all of the people in your household. Can you estimate how much income each person in your household currently earns (take-home income or net) per month from labour related activities?  *Enter:* Estimated current net income from labour related activities of the household per month: | ________ VND | | | *The interviewer should go through the list of people created in Q40, which has the estimated income for each person from Q54. The interviewer should ask if each person on the list is currently working. The interviewer should write down the estimated income from labour related activities for each person. Once the interviewer has obtained an estimated current income for each household member, the interviewer should add the estimated income for each person in the household and enter the total here.* |
| 58 | Can you estimate how much income each person in your household currently earns (take-home/net income) per month from non-labour related activities? | ________ VND | | | *Include income from owning rentals, interest from investments, etc… If no other income, mark 0 VND.* |
| 59. | Did you or your household receive any social welfare payment after you were diagnosed with TB? | 0. No  1. Paid sick leave  2. Disability grant  3. Cash transfer for poor families  4. Other cash transfer | | | *Multi-select* |
| 59a. | -> How much did you receive for paid sick leave in the last month? | ________ VND | | | *Ask if Q59=1* |
| 59b. | -> How much did you receive for the disability grant in the last month? | ________ VND | | | *Ask if Q59=2* |
| 59c. | -> Did study officers from the social support pilot assist you in obtaining a disability grant? | 1. Yes  2. No | | | *Ask if Q59=2* |
| 59d. | -> If yes, how much support did the study officers help you receive from the disability grant in the last month? | ________ VND | | | *Ask if Q59c=1. Yes* |
| 59e. | -> How much did you receive for cash transfers for poor families in the last month? | ________ VND | | | *Ask if Q59=3*  *Include social support pilot payment s(CCT & UCT) and Ho Ngheo payments* |
| 59f. | -> Did the study officers from the social support pilot assist you in obtaining any cash transfers for poor families? | 1. Yes  2. No | | | *Ask if Q59=3* |
| 59g. | -> If yes, how much support did the study officers help you receive from the cash transfers for poor families in the last month? | ________ VND | | | *Ask if Q59f=1. Yes*  *Include all social support (UCT & CCT) payments along with any other cash payments for poor families* |
| 59h. | -> How much did you receive for other cash transfers in the last month? | ________ VND | | | *Ask if Q59=4 Include payments to elderly people and any other cash transfers* |
| 59i. | -> Did the study officers from the social support pilot assist you in obtaining any other cash transfers? | 1. Yes  2. No | | | *Ask if Q59=4* |
| 59j. | -> If yes, how much support did the study officers help you receive from other cash transfers in the last month? | ________ VND | | | *Ask if Q59i=1. Yes* |
| 60. | Do you currently receive vouchers or goods in kind to cope with TB illness? | 1. Yes  2. No | | | *If no, skip to Q61.* |
| 60a. | -> From whom do you receive the voucher/ goods | 1. Government  2. NGO  3. Employer  4. Private donation  5. Other: ________ | | | *Ask if Q60 is 1.Yes*  *Multi-select* |
| 60b. | -> How much do you receive per month in travel vouchers? | ________ VND | | | *Ask if Q60 is 1.Yes* |
| 60c. | -> Did the study officers from the social support pilot assist you in obtaining these travel vouchers? | 1. Yes  2. No | | | *Ask if Q60b >0* |
| 60d. | -> If yes, how much support did the study officers help you receive from travel vouchers in the last month? | ________ VND | | | *Ask if Q60c. is 1.Yes* |
| 60e. | -> How much do you receive per month in food support? | ________ VND | | | *Ask if Q60 is 1.Yes* |
| 60f. | -> Did the study officers from the social support pilot assist you in obtaining this food support? | 1. Yes  2. No | | | *Ask if Q60e >0* |
| 60g. | -> If yes, how much support did the study officers help you receive the food support in the last month? | ________ VND | | | *Ask if Q60f. is 1.Yes* |
| 60.h | -> How much do you receive per month in other enablers? | ________ VND | | | *Ask if Q60 is 1.Yes* |
| 60i. | -> Did the study officers from the social support pilot assist you in obtaining these other enablers? | 1. Yes  2. No | | | *Ask if Q60h >0* |
| 60j. | -> If yes, how much did the study officers help you receive the other enablers in the last month? | ________ VND | | | *Ask if Q60i. is 1.Yes* |
| 61. | How many rooms are there in the house, excluding the bathroom? | ________ Rooms | | | *All spaces in the house are considered rooms, even if used for storage or parking a motorbike. One space = 1 room. Exclude bathrooms.* |
| 62. | Besides yourself, does anyone else of your household receive treatment for TB? | 1. Yes  2. No | | |  |
| 62a. | -> How many people in your household receive treatment for TB? | ________ people | | | Ask if Q60 is 1.Yes |
| 63. | Has the TB illness affected your social or private life in any way? | 1. No  2. Food insecurity  3. Divorce or Separated from spouse/partner  4. Loss of Job  5. Interrupted schooling  6. Social exclusion  7. Isolation from family  8. Experienced stigma  9. Self-limiting contact/communication with others  7. Other________ | | | More than one category allowed. |

|  | ***Coping*** | | | | | | |
| --- | --- | --- | --- | --- | --- | --- | --- |
| 64 | **Did you borrow or receive any money to cover costs incurred since you started TB treatment** | | | | 1. Yes  2. No | *Pawning items should be included here since it is a loan secured by an asset.* | |
| 64a. | **-> If yes, how much did you borrow (in total) or receive?** | | | | ________ VND | *Ask If Q64 is 1.Yes* | |
| 64b. | **-> From whom did you borrow?** | | | | 1. Family  2. Neighbors/friends  3. Private bank  4. Cooperative  5. Employer  6. “Unofficial lender” (Black market)  7. Pawnshop  8. Other: ________ | *Ask If Q64 is 1.Yes*  *Multiple responses allowed.* | |
| 64c. | **-> Do you have the capability to pay the loans back?** | | | | 1. Yes  2. No  3. Some of the loans (if more than one) | *Ask If Q64 is 1.Yes* | |
| 64d. | **-> Have you started paying back the loan(s)?** | | | | 1. Yes  2. No | *Ask If Q64 is 1.Yes* | |
| 64e. | **-> If yes, when did you start?** | | | | 1. Yes, before treatment started  2.Yes, during the Intensive treatment phase | *Ask If* ***Q64c*** *is 1.Yes or 3. Some of the loans* | |
| 64f. | **-> What are the repayment terms of the loan, including interest?** | | | | 1. Weekly repayments  2. Monthly repayments  3. One off repayment  4. Fixed percentage of the loan repayment  5. No fixed payment plan- Whenever money is available | *Ask If Q64 is 1.Yes* | |
| 64g. | **-> Amount per week** | | | | ________ VND | *Ask if 64f is 1. Weekly payments* | |
| 64h. | **-> Amount per month** | | | | ________ VND | *Ask if 64f is 2. Monthly payments* | |
| 64i. | **-> Amount of the one off payment** | | | | ________ VND | *Ask if 64f is 3. One off payment* | |
| 64j. | **-> Amount of the fixed percentage of the loan** | | | | ________ VND | *Ask if 64f is 4. Fixed percentage of the loan* | |
| 64k. | **-> Amount paid back (when no fixed payment plan)** | | | | ________ VND | *Ask if 64f is* 5. No fixed payment plan | |
| 65. | **Have you sold any of your property to finance the costs incurred during TB treatment?** | | | | 1. Yes  2. No |  | |
| 65a. | **-> If yes, what did you sell?** | | | | 1. Land  2. Livestock  3. Transport/vehicle  4. Household item  5. Farm produce  6. Gold/jewelry  7. Other :________ | *Ask if Q65 is 1.Yes*  *Multiple responses allowed. Circle all that are mentioned* | |
| 65b. | **-> How much money did you receive from the sale of all items of your property (in total)?** | | | | ________ VND | *Ask if Q65 is 1.Yes* | |
| 65c. | **The assets that you sold, were they previously supporting the family income (or expenditure)?** | | | | 1. Yes  2. No | *Ask if Q65 is 1.Yes* | |
| 65d. | **-> If yes indicate monthly income previously generated by the assets** | | | | ________ VND | *Ask if Q65 is 1.Yes* | |
| 66. | **What is the estimated market value of all the property you sold?** | | | | ________ VND | *Market value refers to the value that could have been realized if the seller was not under pressure to sell quickly to raise funds.* | |
| 67. | **Thank you for your cooperation! Is there anything you would like to ask or say?** | | | | | |  |
|  | | | | | | |  |
| **68.** | **Comments by Interviewer:** | | | | | |  |
|  | | | | | | |  |
| **Date** *(dd/mm/yyyy)*: | | ____/____/____ | **Signature interviewer:** | ________________________________________________ | | |  |

**Interviewer Guidelines**

**(To be printed and brought to every interview)**

**Fill out checklist before each interview:**

| **Checklist for which parts of the questionnaire to fill for different treatment categories** | | | |
| --- | --- | --- | --- |
| **Treatment category and treatment phase at time of interview** | **Questionnaire part III**  **(tick when filled)** | **Questionnaire part IV**  **(tick when filled)** | **Supervisor check** |
| **Not MDR** | | | |
| First line, new case, interviewed in the intensive treatment phase | *Filled □* | *Filled □* |  |
| First line, new case, interviewed in the continuation treatment phase | *Do not fill* | *Filled □* |  |
| First line, relapse or retreatment | *Do not fill* | *Filled □* |  |
| **MDR** | | | |
| MDR, new case, interviewed in the intensive treatment phase | *Filled □* | *Filled □* |  |
| MDR, new case, interviewed in the continuation treatment phase | *Do not fill* | *Filled □* |  |
| MDR, relapse or re-treatment | *Do not fill* | *Filled □* |  |

**General guidelines:**

1. A duplicate of this signed questionnaire should be offered to the patient.
2. Questions should be read exactly as they appear in the column labeled Question.
3. If the interviewee responds to a question with a range, then the average (mean) of the range should be taken and recorded on the survey. For example, if you ask how for many weeks? And the patient responds with 2-3 weeks. Then the response 2.5 weeks should be entered on the form.

**Health insurance guidelines for Question 14:**

| **Number 1** | SHI covers 100% (without limitation on percentage of reimbursement for some specific/special medicines, chemicals medical supplies and technological services) and transportation to the referral hospitals. | This SHI card goes along with first 2- letter abbreviations **CC, TE.** |
| --- | --- | --- |
| **Number 2** | SHI covers 100% (with limitation on percentage of reimbursement for some specific/special medicines, chemicals medical supplies and technological services) and transportation to the referral hospitals. | This SHI card goes along with first 2- letter abbreviations **CK, CB, KC, HN, DT, DK, XD, BT, TS.** |
| **Number 3** | SHI covers 95% (with limitation on percentage of reimbursement for some specific/special medicines, chemicals medical supplies and technological services). SHI covers 100% of examination and treatment costs at communal hospitals and cost for 1 visit less than 15% of government base salary. | This SHI card goes along with first 2- letter abbreviations **HT, TC, CN** |
| **Number 4** | SHI covers 80% (with limitation on percentage of reimbursement for some specific/special medicines, chemicals medical supplies and technological services). SHI covers 100% of examination and treatment costs at communal hospitals and cost for 1 visit less than 15% of government base salary. | This SHI card goes along with first 2- letter abbreviations **DN, HX, CH, NN, TK, HC, XK, TB, NO, CT, XB, TN, CS, XN, MS, HD, TQ, TA, TY, HG, LS, PV, HS**, **SV, GB, GD.** |
| **Number 5** | SHI covers 100% including costs of examination and treatment outside the scope of reimbursement; costs of transportation. | This SHI card goes along with first 2-letter abbreviations **QN, CA, CY.** |

**Where to find the health insurance number and type on the health insurance card:**

**Instructions for Q17- Pre-treatment costs and Q22 Costs of hospitalizations and emergency care**

- Fill one line per visit in chronological order. The visits should correspond with Q16 exactly.
- For all that don’t apply, mark NA
- If there were payments for an item, but the patient cannot remember the amount, mark NR
- Add more rows if there were more visits than rows.

| Visits | Includes outpatient visits as well as hospitalizations. Should be filled in chronological order, 1st visit=visit 1. |
| --- | --- |
| Provider type | Must correspond to Questions 16 and 21. The provider type is duplicated to ensure that Q16-Q17 and Q21-Q22 matches. |
| Number of days hospitalized | To be filled for each hospitalization. Should be filled in chronological order. If the patient was not hospitalized on this visit, then mark NA. |
| Travel time and time spent for visit | Hours spent to travelling to and from facility. This column measures the total time from leaving home to returning home, including the time waiting for outpatient visits. For hospitalizations, multiply the number of days by 24 hours and include hours.- |
| A1- Daily room charges | Fees for time spent in the hospital. Only for hospitalizations, and only to be filled if not covered by the other cost items (consultation fee, radiography etc.). If the patient was not hospitalized, then fill with NR. |
| A2- Consultation fee | Other charges paid by the patient, not covered under day charge, including direct payment to health care staff. For hospitalizations, include total out-of-pocket payments for the entire stay. |
| A3- Radiography and other imaging | Out-of-pocket payments for imaging investigation (X-rays, CT-scan, ultrasound), TB-specific and other. |
| A4- Lab test fees | Out-of-pocket payments for all tests, TB specific and others. |
| A5- Other procedures | Out-of-pocket payments for biopsy, bronchial lavage etc… but not surgery unrelated to TB. |
| A6- Medicine fees | Any medicine (TB or other) prescribed before TB was diagnosed under NTP. |
| A7- Other, including nutritional supplements | Any other treatments paid out of pocket, such as nutritional supplements medically indicated. This is for all known costs that do not fit within A1-A6. |
| A8 Un-itemized costs | If the patient did not receive a bill or does not know the cost breakdown for medical out-of-pocket payments, then leave A1-A7 blank and fill in column A8 for the estimate of all un-itemized costs. |
| B1- Travel | Out-of-pocket payments for travel to the facility, for both patient and any household member who accompanied the patient. |
| B2- Food | Out-of-pocket payments for additional food bought in relation to travelling the health care visit, and during visit or hospitalization, for both patient and any household member. |
| B3- Other, including accommodation | Includes out-of-pocket payments related to renting a room/bed during health care visits, and any other non-medical payments related to health care visit, for both patient and any household member. |
| B4- Un-itemized costs | If the patient cannot distinguish between the costs for categories B1-B3, but can estimate the total, then leave B1-B3 blank and fill out B4. |
| On this visit did you use health insurance? | Circle Yes, if the patient has health insurance (Q14) and the health insurance and submitted the health insurance information to the facility at the beginning of the visit. Circle No, if the patient does not have health insurance (Q14) or has health insurance and did not submit the information to the facility. Also circle No if the patient was at a medical facility where their insurance was not valid. |
| Reimbursement from health insurance | Amount reimbursed to patient through medical insurance (private or social security) so far, does not include expected future reimbursement. This is not the amount the health insurance pays on the patient’s behalf- it is the money that the health insurance company returns to the patient. |

**Template for establishing household size and income in Questions 54, 55, 57 and 58.**

**Definition of a household:** *People who live under the same roof as the patient, share a kitchen and have been living there for at least one month.*

**Include the patient as a member of the household.**

| **Number** | **Person identifier** | **Under 15 (X)** | **Over 15(X)** | **Question 53- estimated monthly net income from labor related activities before TB**  *(for total add all amounts in this column)* | **Question 54- estimated monthly net income from non-labor related activities before TB**  *(for total add all amounts in this column)* | **Question 57 estimated monthly net income from labor related activities after TB**  *(for total add all amounts in this column)* | **Question 58- estimated monthly net income from non-labor related activities after TB**  *(for total add all amounts in this column)* |
| --- | --- | --- | --- | --- | --- | --- | --- |
| **1** |  |  |  |  |  |  |  |
| **2** |  |  |  |  |  |  |  |
| **3** |  |  |  |  |  |  |  |
| **4** |  |  |  |  |  |  |  |
| **5** |  |  |  |  |  |  |  |
| **6** |  |  |  |  |  |  |  |
| **7** |  |  |  |  |  |  |  |
| **8** |  |  |  |  |  |  |  |
| **9** |  |  |  |  |  |  |  |
| **10** |  |  |  |  |  |  |  |
|  | **Total** |  |  |  |  |  |  |

**Interviews 2 and 3 use the following survey form:**

Interview 2 or 3 survey form:

***Part I- Costs during the current phase of TB treatment***

| 1 | ***Since the last interview*, did you seek care or advice for your symptoms of the current illness at either hospitals, commune health stations, district TB units, district health centres, pharmacies, or a private doctor?** | 1. Yes  2. No | *Yes 🡪 Fill out table for Q1a and Q1b*  *No 🡪 Skip to Q4 on page 4* |
| --- | --- | --- | --- |
|  | **-> We would like to construct a timeline for every visit that you made to different providers for the symptoms of your current illness*.*** *(Enter in chronological order in 1a and 1b)* | **1a. Provider type**   20. Commune Health Station 21. District TB Unit 22. District Health Centre 23. Pharmacy/Drugstore 24. Private clinic 25. Public hospital where the patient is registered 26. Public hospital- **not** where the patient is registered 27. Private hospital 28. Herbalist/traditional practitioners 29. Other__________________________ | **1b. How many weeks since the last interview did you visit each of these providers?** |
|  | **1^st^ visit** | Provider category: ________ | Number of weeks since last interview: ________ |
|  | **2^nd^ visit** | Provider category: ________ | Number of weeks since last interview: ________ |
|  | **3^rd^ visit** | Provider category: ________ | Number of weeks since last interview: ________ |
|  | **4^th^ visit** | Provider category: ________ | Number of weeks since last interview: ________ |
|  | **5^th^ visit** | Provider category: ________ | Number of weeks since last interview: ________ |
|  | **6^th^ visit** | Provider category: ________ | Number of weeks since last interview: ________ |
|  | **7^th^ visit** | Provider category: ________ | Number of weeks since last interview: ________ |
|  | **8^th^ visit** | Provider category: ________ | Number of weeks since last interview: ________ |
|  | **9^th^ visit** | Provider category: ________ | Number of weeks since last interview: ________ |
|  | **10^th^ visit** | Provider category: ________ | Number of weeks since last interview: ________ |

| 2. | **Are you currently hospitalized?** | 1. Yes  2. No | If yes, cost data collected applies to the first row of Q3b  If no, ask Q4. |
| --- | --- | --- | --- |
| 3. | **Since the last interview, have you been hospitalized or sought emergency care because of TB?** | 1. Yes  2. No | *If answer to questions 1 to 3 are “no”, then skip to Q4.* |
| 3a. | **-> If yes, how many times?** | ______ Times |  |
| **Instructions for the table in Q3b.**   - See table below, and ask for each item. Fill one line per visit. - The table should be filled in chronological order. - For all that don’t apply, mark/select NA - If there were payments for an item, but the patient cannot remember the amount, mark NR   Explanation of table headings:   - Type of hospital: fill in provider type according to categories in Q1a - Number of days hospitalized: Include the current day if Q2 is Yes. Must be filled out for each hospitalization. Should be filled in chronological order. - Travel time and time spent for visit: Hours spent travelling to and from facility. This column measures the total time from leaving home to returning home, including the time waiting for outpatient visits. For hospitalizations, multiply the number of days by 24 hours and include hours. - A1- Daily room charges: Fees for time spent in the hospital. Only for hospitalizations, and only to be filled if not covered by the other cost items (consultation fee, radiography etc.). If the patient was not hospitalized, then fill with NR - A2- Consultation fee: Other charges paid by the patient, not covered under day charge, including direct payment to health care staff. For hospitalizations, include total out-of-pocket payments for the entire stay - A3- Radiography and other imaging: Any imaging investigation (x-rays, CT-scan, ultrasound), TB-specific and other - A4- Lab test fees: includes all tests, TB specific and others, including cost of transporting samples, if paid by patient - A5- Other procedures: includes biopsy, bronchial lavage, etc. but not surgery unrelated to TB - A6- Medicines (do not include TB medicines): The total cost of medicines that are not TB medications. - A7- TB medicines: fees for TB medicines only, bought inside or outside hospital. Do not include the value of free medicines. - A8- Other medicines, including nutritional supplements: any other medicine, including nutritional supplements. This is for all known costs that do not fit within A1-A8. - A9- Un-itemized medical payments- If the patient did not receive a bill or does not know the cost breakdown for medical out-of-pocket payments, then leave A1-A7 blank and fill in column A8 for the estimate of all un-itemized costs. - B1- Travel: out-of-pocket payment for travel to the facility (does not include income loss), for both patient and any household member who accompanied the patient. - B2Food: Out-of-pocket payments for additional food bought in relation to travelling the health care visit, and during visit or hospitalization, for both patient and any household member. - B3- Other, including accommodation: Includes out-of-pocket payments related to renting a room/bed during health care visits, and any other non-medical payments related to health care visit, for both patient and any household member. - B4- Un-itemized Non-medical out of pocket payments: If the patient cannot distinguish between the costs for categories B1-B3, but knows the total, then leave B1-B3 blank and fill B4. - On this visit did you use health insurance? Circle Yes, if the patient has health insurance and the health insurance and submitted the health insurance information to the facility at the beginning of the visit. Circle No, if the patient does not have health insurance or has health insurance and did not submit the information to the facility. Also circle No if the patient was at a medical facility where their insurance was not valid - C- Health insurance reimbursement: Amount reimbursed to patient through medical insurance (private or social security) so far, does not include expected future reimbursement. This is not the amount the health insurance pays on the patient’s behalf- it is the money that the health insurance company returns to the patient | | | |

**3b. About how much money and time did you spend for each of these hospitalizations/emergency care? (For patient, guardian and the person accompanying the Patient)**

|  | | | |  | Medical out-of-pocket payments,  (Total per visit)  (A) | | | | | | | | Non-medical out-of-pocket payments,  (Total per visit)  (B) | | | | | Health insurance contribution | | |
| --- | --- | --- | --- | --- | --- | --- | --- | --- | --- | --- | --- | --- | --- | --- | --- | --- | --- | --- | --- | --- |
| Hospitalization or emergency care | Type of hospital (see Q1a) | # of days hospitalized (for hospitalizations only) | Travel time and time spent for visit (Hours) | Daily room charges  (for hospitalizations only)  A1 | Consultation fee  (total for stay)  A2 | Radiography and other imaging  A3 | Lab  tests  A4 | Other procedure  A5 | Medicines  (do not include TB medicines)  A6 | TB medicines (do not include free meds)  A7 | Other medicines, incl nutritional supple-ments  A8 | Un-itemized medical payments  A9 | Travel  B1 | Food during hospital stay  B2 | Other, including accommo-dation  B3 | Un-itemized Non-medical out-of-pocket payments  B4 | On this visit did you use health insurance? | | Health insurance reimbursement to patient  (C) |  |
| 1st |  |  |  |  |  |  |  |  |  |  |  |  |  |  |  |  | Y/N | |  |  |
| 2nd |  |  |  |  |  |  |  |  |  |  |  |  |  |  |  |  | Y/N | |  |  |
| 3rd |  |  |  |  |  |  |  |  |  |  |  |  |  |  |  |  | Y/N | |  |  |
| 4th |  |  |  |  |  |  |  |  |  |  |  |  |  |  |  |  | Y/N | |  |  |
| 5th |  |  |  |  |  |  |  |  |  |  |  |  |  |  |  |  | Y/N | |  |  |
| 6th |  |  |  |  |  |  |  |  |  |  |  |  |  |  |  |  | Y/N | |  |  |
| 7th |  |  |  |  |  |  |  |  |  |  |  |  |  |  |  |  | Y/N | |  |  |
| 8th |  |  |  |  |  |  |  |  |  |  |  |  |  |  |  |  | Y/N | |  |  |
| 9th |  |  |  |  |  |  |  |  |  |  |  |  |  |  |  |  | Y/N | |  |  |
| 10th |  |  |  |  |  |  |  |  |  |  |  |  |  |  |  |  | Y/N | |  |  |

|  | ***Part II: Costs for DOT and food costs during ambulatory care***  ***(****DOT (Directly observed treatment) visit is for the supervision of daily intake of medicines, i.e, what is done every day. These questions are not referring to less frequent trips to pick up drugs (e.g., weekly), which are explored from Q5 onwards.)* | | | |
| --- | --- | --- | --- | --- |
| 4. | **On a daily basis, do you currently take your medicines yourself without supervision or support (self-administered) or do you have a treatment supervisor or supporter (DOT)?** | 1. Self-administered  2. DOT via DTU  3. DOT via DTU/commune health station | - *If 1. self-administered skip to Q5* - *If 2. DOT via DTU or 3. DTU/commune health station, ask Q4a- Q4g* |  |
| 4a. | **-> For any form of DOT, how many times a week?** | _______ Times/week | *The maximum will be 7 times a week* |  |
| 4b. | **-> Who is your DOT provider/supporter?** | 1. Commune health station  2. DTU officer  3. Community health workers (CTV) or TB counsellors (TVV)  4. Work place  5. Family member  6. Other_______________ |  |  |
| 4c. | **-> How long did the last DOT visit take, including travel time and waiting time (total turnaround time)?** | ________Minutes |  |  |
| 4d. | **-> What was the cost of transport (return) for the last DOT visit, including parking costs, in total for you and any accompanying household member?** | ________ VND |  |  |
| 4e. | **-> How much did you spend on food, drinks and accommodation for the last DOT visit (on the road, while waiting, lunch etc.), in total for you and any accompanying household member?** | ________ VND |  |  |
| 4f. | **-> If there were any other fees or costs, how much did you spend on other fees during your last DOT visit?** | ________ VND | *Include: plastic bags for sputum transport, cost of injections, travel for a DTU officer to the patient’s home, etc..*  *If no other costs, then answer 0 VND* |  |
| 4g. | **-> Did somebody in your household accompany you for your last DOT visit?** | 1. Yes  2. No |  |  |

|  | ***Part III: Costs of picking up drugs and food costs during outpatient care***  *This section does not concern DOT visits, which should have recorded in the last section, but should filled if patient or other household member picks up drugs for either bringing to DOT provider or for self-administered treatment.* | | | |
| --- | --- | --- | --- | --- |
| 5. | **Do you or a household member pick up TB drugs (for self-administered treatment or to bring to your DOT supervisor/supporter)?** | 1. Yes  2. No | *If patient is on DOT and patient or household member is* ***not*** *picking up drugs to bring to DOT provider, then the answer is no.*  *If yes, then answer 5a.-5h.*  *If no, skip to Q6 on page 6* |  |
| 5a. | **-> How often do you or a household member pick up TB drugs in the current treatment phase?** | 1. Every day  2. Every week  3. Every 2 weeks  4. Every month  5. Other____________ |  |  |
| 5b. | **->Where do you or your household member pick up your TB drugs?** | 1. Commune health station  2. District TB unit  3. Other:________ | *If the patient has visited different places, tick the most recent one.* |  |
| 5c. | **-> What accommodation cost did you and any accompanying household member have when you last picked up drugs?** | ________ VND |  |  |
| 5d. | **-> How long did the last visit to pick up drugs take, including travel time and waiting time (total turnaround time)?** | ________Minutes |  |  |
| 5e. | **-> What was the cost of transport (return) last time you picked up drugs, including parking costs, in total for you and any accompanying household member?** | ________ VND |  |  |
| 5f. | **-> How much did you spend on food and drinks last time you picked up drugs (on the road, while waiting, lunch etc.), in total for you and any accompanying household member?** | ________ VND |  |  |
| 5g. | **-> If there were any other fees or costs, how much did you spend on these other fees the last time you picked up drugs, in total, for you and any accompanying household member?** | ________ VND | *If no other costs, then answer 0 VND* |  |
| 5h. | **-> Did somebody in your household accompany you for your last visit to pick up drugs or did they pick up drugs for you?** | 1. Yes  2. No |  |  |

|  | ***Part IV: Cost during outpatient visits for medical follow-up (see the doctor or nurse, have tests)*** | | |
| --- | --- | --- | --- |
| 6. | **How many TB-related medical follow-up visits have you had since the last interview (to see the doctor or nurse, have follow- up tests, etc.)?** | ________ Times | *This concerns clinical check-up, follow up, and additional visits due to side effects or other TB related issues. It does not include DOT visits or visits to pick up drugs.* |
| 7. | **How long did the last follow-up medical outpatient visit take, including travel time and waiting time (total turnaround time)?** | ________ Minutes |  |
| 8. | **What was the cost of transport (return) at the last follow-up medical outpatient visit, including parking, in total for you and any accompanying household member?** | ________ VND | *Cost related to the latest visit. If the interview takes place at the end of such a visit use the costs for the present visit for Q8-18.* |
| 9. | **How much did you spend on food and drinks at the last follow-up medical outpatient visit (on the road, while waiting, lunch etc.), in total for you and any accompanying household member?** | ________ VND |  |
| 10. | **What accommodation cost did you have for the last follow-up medical outpatient visit, in total, for you and any accompanying household member?** | ________ VND |  |
| 11. | **What fees did you pay during your last follow-up medical outpatient visit for registration/consultation?** | ________ VND |  |
| 12. | **What fees did you pay during your last follow-up medical outpatient visit for radiography and other imaging?** | ________ VND |  |
| 13. | **What fees did you pay during your last follow-up medical outpatient visit for tests, TB tests and others?** | ________ VND |  |
| 14. | **What fees did you pay during your last follow-up medical outpatient visit for other procedures?** | ________ VND |  |
| 15. | **What fees did you pay at your last follow-up medical outpatient visit for TB medicines, including prescriptions for medicines bought outside the facility?** | ________ VND |  |
| 16. | **What fees did you pay during your last follow-up medical outpatient visit for other medicines, including nutritional supplements?** | ________ VND |  |
| 17. | **What other fees not listed in the previous questions did you pay during your last follow-up medical outpatient visit?** | ________ VND | *Examples include: masks, health staff fee (unofficial)* |
| 18. | **Did somebody in your household accompany you for your last medical follow-up visit?** | 1. Yes  2. No |  |

|  | ***Part V: Costs for nutritional/food supplements*** | | | | | | | |
| --- | --- | --- | --- | --- | --- | --- | --- | --- |
| 19. | **Do you buy any nutritional supplements outside your regular diet because of the TB illness, for example vitamins, meat, energy drinks, or fruits as recommended by health care staff?** | | | 1. Yes  2. No | | *If yes, ask Q19a*  *If no, skip to Q20* | | |
| 19a. | **-> How much did you spend on nutritional supplements (vitamins, meat, energy drinks, or fruits) in the past week approximately?** | | | ________ VND | |  | | |
|  | ***Part VI: Time loss for guardians***   - ***Not to be filled if the patient is under 15 years – for children, all questions concerning costs, time spent, income, and income loss concern cost for the guardian.*** - ***Note: out-of-pocket costs of transport, food, accommodation for guardian should be included in questions on Part VI.*** | | | | | | | |
| 20. | Earlier in the survey, you indicated that others have accompanied you as you sought care for TB and we collected the out-of-pocket costs for the person who accompanied you**. Can you identify the last time someone accompanied you as you sought healthcare and the type of visit that you were going for?** | | | 0. No one has accompanied me  1. Hospitalization  2. DOT visit  3. Drug pick-up  4. Medical follow-up visit | | | *If yes, to Q4g, Q5h or 18 then responses to Q20 should be 1-4.*  *If Q20 is 0. No one accompanied me, then skip to Q21* | |
| 20a. | **->Who accompanied you to that visit?** | | | 1. Grandfather  2. Grandmother  3. Father  4. Mother  5. Uncle  6. Aunt  7. Husband  8. Wife  9. Brother  10. Sister  11. Cousin (male)  12. Cousin (female)  13. Child (son)  14. Child (daughter)  15. Friend  16. Other household member_____ | | |  | |
| 20b. | **->During that last visit, did the person who accompanied you, take time off work?** | | | 1. Yes  2. No | | |  | |
| 20c. | **->Did that person lose any income as they accompanied you for the visit?** | | | 1. Yes  2. No | | |  | |
| 20d. | **->How much income would you estimate that the person who accompanied you lost during your last visit?** | | | ________ VND | | | *If Q20c is 1. Yes, then ask 20d.*  *If Q20c is 2. No, then skip to Q21* | |
|  | | ***Part VII: Income (reported) & Income changes & social consequences*** | | | | | |  |
| 21. | | **What is your main occupation now?** | 14. School student 15. Technician 16. Service 17. Factory worker 18. Farmer 19. Government employee 20. Teacher 21. Retiree 22. Homemaker 23. Unemployed 24. Manual labor 25. Microenterprise owner   Other (specify): _______ | | *If patient is under 15 years, this question is for the guardian.* | | |  |
| 22. | | **What is your primary employment now?** | 0. Unemployed  1. Formal paid work  2. Informal paid work  3. Retired  4. Student  5. Housework  6. Other (specify): _____ | | *If patient is under 15 years, this question is for the guardian.*  *“3.Retired” is defined as when a person is no longer working because of their age. It does not only refer to government employees who receive a pension.* | | |  |
| 23. | | **Are you currently working?** | 1. Yes  2. No | | *If Q23 is 1.Yes, then ask Q23a-23c.*  *If Q23 is 2.No, then ask Q23d -23f.* | | |  |
| 23a. | | **-> If yes, how many hours per week are you working now?** | ________ hours/week | | *Ask if Q23 is 1.Yes* | | |  |
| 23b. | | **-> If yes, what is your current monthly take-home income (net)?** | ________ VND | | *Ask if Q23 is 1.Yes* | | |  |
| 23c. | | **-> If yes, how many working days have you lost because of TB since our last interview?** | ________ Days | | *Ask if Q23 is 1.Yes*  *Working days: e.g., if a patient was not able to work for 5 half days and lost income for these, the number of days lost is 0.5*5=2.5.* | | |  |
| 23d. | | **-> If no, when did you stop working?** | ____/____/____ | | *Ask if Q23 is 2. No*  *Day/month/year* | | |  |
| 23e. | | **-> If no, why did you stop working?** | 1.Illness related to TB  2. Not related to TB | | *Ask if Q23 is 2. No* | | |  |
| 23f. | | **-> If no, how many working days did you lose before you stopped working entirely?** | ________ Days | | *Ask if Q23 is 2. No*  *Working days: e.g., if a patient was not able to work for 5 half days and lost income for these, the number of days lost is 0.5*5=2.5. Report for all days before job loss.* | | |  |
| 24. | | We are interested in understanding the current income of your household. We previously listed all of the people in your household during the first interview. Can you estimate how much income each person in your household currently earns (take-home income or net) per month from labour related activities?  *Enter:* Estimated current net income from labour related activities of the household per month: | ________ VND | | *The interviewer should go through the list of people created in the first interview, which has the estimated income for each person from Q54 in the first interview The interviewer should ask if each person on the list is currently working. The interviewer should write down the estimated income from labour related activities for each person. Once the interviewer has obtained an estimated current income for each household member, the interviewer should add the estimated income for each person in the household and enter the total here.* | | |  |
| 25 | | Can you estimate how much income each person in your household currently earns (take-home/net income) per month from non-labour related activities? | ________ VND | | *Include income from owning rentals, interest from investments, etc… If no other income, mark 0 VND.* | | |  |
| 26. | | Did you or your household receive any social welfare payment after you were diagnosed with TB? | 0. No  1. Paid sick leave  2. Disability grant  3. Cash transfer for poor families  4. Other cash transfer | | *Multi-select* | | |  |
| 26a. | | -> How much did you receive for paid sick leave in the last month? | ________ VND | | *Ask if Q26=1, Paid Sick Leave* | | |  |
| 26b. | | -> How much did you receive for the disability grant in the last month? | ________ VND | | *Ask if Q26=2. Disability Grant* | | |  |
| 26c. | | -> Did study officers from the social support pilot assist you in obtaining a disability grant? | 1. Yes  2. No | | *Ask if Q26=2. Disability Grant* | | |  |
| 26d. | | -> If yes, how much support did the study officers help you receive from the disability grant in the last month? | ________ VND | | *Ask if Q26c=1. Yes* | | |  |
| 26e. | | -> How much did you receive for cash transfers for poor families in the last month? | ________ VND | | *Ask if Q26=3. Cash transfer for poor families*  *Include social support pilot payment s(CCT & UCT) and Ho Ngheo payments* | | |  |
| 26f. | | -> Did the study officers from the social support pilot assist you in obtaining any cash transfers for poor families? | 1. Yes  2. No | | *Ask if Q26=3. Cash transfer for poor families* | | |  |
| 26g. | | -> If yes, how much support did the study officers help you receive from the cash transfers for poor families in the last month? | ________ VND | | *Ask if Q26f=1. Yes*  *Include all social support (UCT & CCT) payments along with any other cash payments for poor families* | | |  |
| 26h. | | -> How much did you receive for other cash transfers in the last month? | ________ VND | | *Ask if Q26=4. Other cash transfers*  *Include payments to elderly people and any other cash transfers* | | |  |
| 26i. | | -> Did the study officers from the social support pilot assist you in obtaining any other cash transfers? | 1. Yes  2. No | | *Ask if Q26=4 Other cash transfers* | | |  |
| 26j. | | -> If yes, how much support did the study officers help you receive from other cash transfers in the last month? | ________ VND | | *Ask if Q26i=1. Yes* | | |  |
| 27. | | Do you currently receive vouchers or goods in kind to cope with TB illness? | 1. Yes  2. No | | *If no, skip to Q28.* | | |  |
| 27a. | | -> From whom do you receive the voucher/ goods | 1. Government  2. NGO  3. Employer  4. Private donation  5. Other: ________ | | *Ask if Q27 is 1.Yes*  *Multi-select* | | |  |
| 27b. | | -> How much do you receive per month in travel vouchers? | ________ VND | | *Ask if Q27 is 1.Yes* | | |  |
| 27c. | | -> Did the study officers from the social support pilot assist you in obtaining these travel vouchers? | 1. Yes  2. No | | *Ask if Q27b >0* | | |  |
| 27d. | | -> If yes, how much support did the study officers help you receive from travel vouchers in the last month? | ________ VND | | *Ask if Q27c. is 1.Yes* | | |  |
| 27e. | | -> How much do you receive per month in food support? | ________ VND | | *Ask if Q27 is 1.Yes* | | |  |
| 27f. | | -> Did the study officers from the social support pilot assist you in obtaining this food support? | 1. Yes  2. No | | *Ask if Q27e >0* | | |  |
| 27g. | | -> If yes, how much support did the study officers help you receive the food support in the last month? | ________ VND | | *Ask if Q27f. is 1.Yes* | | |  |
| 27.h | | -> How much do you receive per month in other enablers? | ________ VND | | *Ask if Q27 is 1.Yes* | | |  |
| 27i. | | -> Did the study officers from the social support pilot assist you in obtaining these other enablers? | 1. Yes  2. No | | *Ask if Q27h >0* | | |  |
| 27j. | | -> If yes, how much did the study officers help you receive the other enablers in the last month? | ________ VND | | *Ask if Q27i. is 1.Yes* | | |  |
| 28. | | How many people are in your household? | ______ people | | *The interviewer should review the list the members of the household from the first interview and assess whether the household size has changed since the last interview.* | | |  |
| 28a. | | -> Number of adults | ________ Adults | |  | | |  |
| 28b. | | -> Number of children | ________ Children | |  | | |  |
| 29. | | Besides yourself, does anyone else of your household receive treatment for TB? | 1. Yes  2. No | | Active TB treatment only. Do not include LTBI treatment. | | |  |
| 29a. | | -> How many people in your household receive treatment for TB? | ________ people | | Ask if Q29 is 1.Yes | | |  |
| 30. | | Has the TB illness affected your social or private life in any way? | 1. No  2. Food insecurity  3. Divorce or Separated from spouse/partner  4. Loss of Job  5. Interrupted schooling  6. Social exclusion  7. Isolation from family  8. Experienced stigma  9. Self-limiting contact/communication with others  10. Other________ | | More than one category allowed. | | |  |

|  | ***Part VIII: Coping*** | | | | | | |
| --- | --- | --- | --- | --- | --- | --- | --- |
| 31. | **Did you borrow or receive any money to cover costs incurred since the last interview?** | | | | 1. Yes  2. No | *Pawning items should be included here since it is a loan secured by an asset.*  *If Q31. Is 1. Yes, ask Q31a.-31k.*  *If Q31 is 2. No, then skip to Q32.* | |
| 31a. | **-> If yes, how much did you borrow (in total) or receive?** | | | | ________ VND | *Ask If Q*31 *is 1.Yes* | |
| 31b. | **-> From whom did you borrow?** | | | | 1. Family  2. Neighbors/friends  3. Private bank  4. Cooperative  5. Employer  6. “Unofficial lender” (Black market)  7. Pawnshop  8. Other: ________ | *Ask If Q*31 *is 1.Yes*  *Multiple responses allowed.* | |
| 31c. | **-> Do you have the capability to pay the loans back?** | | | | 1. Yes  2. No  3. Some of the loans (if more than one) | *Ask If Q*31 *is 1.Yes* | |
| 31d. | **-> Have you started paying back the loan(s)?** | | | | 1. Yes  2. No | *Ask If Q*31 *is 1.Yes* | |
| 31e. | **-> If yes, when did you start?** | | | | 1. Yes, before treatment started  2.Yes, during the Intensive treatment phase  3. Yes, during the continuation treatment phase | *Ask* ***If Q*31*c*** *is 1.Yes or 3. Some of the loans* | |
| 31f. | **-> What are the repayment terms of the loan, including interest?** | | | | 1. Weekly repayments  2. Monthly repayments  3. One off repayment  4. Fixed percentage of the loan repayment  5. No fixed payment plan- Whenever money is available | *Ask If Q*31 *is 1.Yes* | |
| 31g. | **-> Amount per week** | | | | ________ VND | *Ask if* 31*f is 1. Weekly payments* | |
| 31h. | **-> Amount per month** | | | | ________ VND | *Ask if* 31*f is 2. Monthly payments* | |
| 31i. | **-> Amount of the one off payment** | | | | ________ VND | *Ask if* 31*f is 3. One off payment* | |
| 31j. | **-> Amount of the fixed percentage of the loan** | | | | ________ VND | *Ask if* 31*f is 4. Fixed percentage of the loan* | |
| 31k. | **-> Amount paid back (when no fixed payment plan)** | | | | ________ VND | *Ask if* 31*f is* 5. No fixed payment plan | |
| 32. | **Have you sold any of your property to finance the costs incurred during TB treatment?** | | | | 1. Yes  2. No | *If 1. Yes, then ask Q 32a-32e.*  *If 2.No, then skip to Q33.* | |
| 32a. | **-> If yes, what did you sell?** | | | | 1. Land  2. Livestock  3. Transport/vehicle  4. Household item  5. Farm produce  6. Gold/jewelry  7. Other :________ | *Ask if Q*32 *is 1.Yes*  *Multiple responses allowed. Circle all that are mentioned* | |
| 32b. | **-> How much money did you receive from the sale of all items of your property (in total)?** | | | | ________ VND | *Ask if Q*32 *is 1.Yes* | |
| 32c. | **The assets that you sold, were they previously supporting the family income (or expenditure)?** | | | | 1. Yes  2. No | *Ask if Q*32 *is 1.Yes* | |
| 32d. | **-> If yes indicate monthly income previously generated by the assets** | | | | ________ VND | *Ask if Q*32 *is 1.Yes* | |
| 32e. | **What is the estimated market value of all the property you sold?** | | | | ________ VND | *Market value refers to the value that could have been realized if the seller was not under pressure to sell quickly to raise funds.* | |
| 33. | **Thank you for your cooperation! Is there anything you would like to ask or say?** | | | | | |  |
|  | | | | | | |  |
| **34.** | **Comments by Interviewer:** | | | | | |  |
|  | | | | | | |  |
| **Date** *(dd/mm/yyyy)*: | | ____/____/____ | **Signature interviewer:** | ________________________________________________ | | |  |
