# Supplementary material for: Why condition cash transfers? A multimethod pilot study evaluating the conditioning of cash transfers for tuberculosis treatment
Source: BMC Health Serv Res. 2026 Jul 30;26:1056. doi: 10.1186/s12913-026-15248-w (PMC13439919; doi:10.1186/s12913-026-15248-w)
Supplement: Supplementary file 3 [file 12913_2026_15248_MOESM3_ESM.docx]

# **Supplementary File 3: Calculating catastrophic costs**

The following formula was used to calculate the TB-related cost burden:

**Definitions**

**Direct medical costs**- Fees for consultations, diagnostic tests, hospitalizations and drugs.

**Direct non-medical costs**- The food, travel and accommodation costs associated with healthcare visits.

**Indirect costs-** Household income lost as a result of TB treatment, using the human capital approach which is calculated by multiplying lost working hours lost by a pre-treatment hourly rate. Time and income loss for both the TB-affected person and care-givers was included in the analysis.

**Cash transfers-** TB-specific cash delivered by the pilot to participants.

**Annual household income**- Self-reported annual household income before TB treatment in USD, imputed based on assets when missing.

**Pre-treatment costs-** The sum of direct and indirect costs incurred between the onset of TB symptoms and receipt of confirmed TB diagnosis.

**Treatment costs**- The sum of direct and indirect costs incurred between confirmed TB diagnosis to completion of TB treatment.

**Total costs-** The sum of pre- and during-treatment costs.

**TB-related cost burden-** Total TB-related costs expressed as a percentage of annual household income.
