# Supplementary material for: Why condition cash transfers? A multimethod pilot study evaluating the conditioning of cash transfers for tuberculosis treatment
Source: BMC Health Serv Res. 2026 Jul 30;26:1056. doi: 10.1186/s12913-026-15248-w (PMC13439919; doi:10.1186/s12913-026-15248-w)
Supplement: Supplementary file 4 [file 12913_2026_15248_MOESM4_ESM.docx]

|  | **Total Observation Time** | | **Time for CCT Cohort**  (Districts Binh Chanh & Go Vap) | | **Time for UCT Cohort**  (Districts 8 & Hoc Mon) | | **Multitasking /**  **supporting both cohorts** | |
| --- | --- | --- | --- | --- | --- | --- | --- | --- |
|  | **Hrs** | **%TOT** (95% CI) | **Hrs** | **%TOT** (95% CI) | **Hrs** | **%TOT** (95% CI) | **Hrs** | **%TOT** (95% CI) |
|  |  |  |  |  |  |  |  |  |
| **Intervention** | **91.7** | **12.1%** (11.8-12.5) | **47.1** | **6.2%** (6.0%-6.5%) | **39.7** | **5.3%** (5.1%-5.5%) | **5.0** | **0.7%** (0.6%-0.7%) |
| Cash transfer preparation | 5.0 | 0.7% (0.6-0.7) | 1.6 | 0.2% (0.2%-0.3%) | 2.8 | 0.4% (0.3%-0.4%) | 0.7 | 0.1% (0.1%-0.1%) |
| Cash transfer distribution | 51.9 | 6.9% (6.6-7.1%) | 32.4 | 4.3% (4.1%-4.5%) | 16.0 | 2.1% (2.0%-2.2%) | 3.5 | 0.5% (0.4%-0.5%) |
| Participant counseling | 34.8 | 4.6% (4.4-4.8%) | 13.1 | 1.7% (1.6%-1.9%) | 21.0 | 2.8% (2.6%-2.9%) | 0.8 | 0.1% (0.1%-0.1%) |
|  |  |  |  |  |  |  |  |  |
| **Administration** | **627.1** | **83.0%** (82.7-83.4%) | **38.7** | **5.1%** (4.9%-5.3%) | **29.1** | **3.9%** (3.7%-4.0%) | **559.3** | **74.1%** (73.7%-74.5%) |
| Travel between sites | 96.1 | 12.7% (12.4%-13.0%) | 30.0 | 4.0% (3.8%-4.2%) | 23.0 | 3.0% (2.9%-3.2%) | 43.2 | 5.7% (5.5%-5.9%) |
| Data management | 47.3 | 6.3% (6.0%-6.5%) | 6.1 | 0.8% (0.7%-0.9%) | 5.1 | 0.7% (0.6%-0.8%) | 36.1 | 4.8% (4.6%-5.0%) |
| Administrative activities | 134.3 | 17.8% (17.4%-18.1%) | 0.7 | 0.1% (0.1%-0.1%) | 0.6 | 0.1% (0.1%-0.1%) | 133.0 | 17.6% (17.3%-18.0%) |
| Other tasks | 135.5 | 17.9% (17.6%-18.3%) | 1.9 | 0.3% (0.2%-0.3%) | 0.5 | 0.1% (0.0%-0.1%) | 133.2 | 17.6% (17.3%-18.0%) |
| Team meetings | 98.3 | 13.0% (12.7%-13.3%) | 0.0 | - | 0.0 | - | 98.3 | 13.0% (12.7%-13.3%) |
| Breaks | 115.6 | 15.3% (15.0%-15.6%) | 0.0 | - | 0.0 | - | 115.6 | 15.3% (15.0%-15.6%) |
|  |  |  |  |  |  |  |  |  |
| **Research** | **36.3** | **4.8%** (4.6%-5.0%) | **12.8** | **1.7%** (1.6%-1.8%) | **7.8** | **1.0%** (0.9%-1.1%) | **15.7** | **2.1%** (2.0%-2.2%) |
|  |  |  |  |  |  |  |  |  |
| *Hrs = Hours; TOT = Total observation time; 95% CI = 95% confidence interval; CCT = conditional cash transfer; UCT = unconditional cash transfer* | | | | | | | | |

# **Additional File 4: Time in motion data with confidence intervals**
