# Supplementary material for: Why condition cash transfers? A multimethod pilot study evaluating the conditioning of cash transfers for tuberculosis treatment
Source: BMC Health Serv Res. 2026 Jul 30;26:1056. doi: 10.1186/s12913-026-15248-w (PMC13439919; doi:10.1186/s12913-026-15248-w)
Supplement: Supplementary file 5 [file 12913_2026_15248_MOESM5_ESM.docx]

# **Additional File 5: Catastrophic costs incurred**

| **Median time loss due to TB** | | | | | | |
| --- | --- | --- | --- | --- | --- | --- |
| **Treatment period** | **Time category** | | **Total**  **(n=108)** | **CCT cohort**  **(n=52)** | **UCT cohort**  **(n=56)** | **p-value** |
| Pre-treatment | Median total hours (IQR) | | 40 (14-114) | 72 (14-116) | 29 (13-111) | 0.257 |
| Treatment | Drug pick-ups | | 360 (222-612) | 365 (219-578) | 360 (226-632) | 0.981 |
|  | Treatment follow-up visits | | 150 (90-300) | 173 (91-300) | 120 (90-270) | 0.329 |
|  | Hospitalization/ emergency care/ unplanned outpatient visit | | 3 (0.5-15) | 3 (1-20) | 3 (0-13) | 0.433 |
|  | Median total hours | | 580 (377-905) | 595 (408-928) | 555 (341-897) | 0.518 |
| Total | Total median hours lost due to TB (IQR) | | 686 (411-954) | 719 (453-1,047) | 686 (412-954) | 0.423 |
| **Median Costs of TB care** | | | | | | |
| **Treatment period** | **Cost category** | | **Total Costs** | **CCT Cohort** | **UCT Cohort** | **p-value** |
| Pre-treatment in USD | Direct medical (IQR) | | 111 (54-171) | 126 (56-195) | 99 (54-136) | 0.167 |
|  | Direct non-medical | | 12 (5-26) | 11 (4-29) | 13 (5-24) | 0.952 |
|  | Indirect costs | | 28 (6-122) | 54 (7-130) | 14 (4-74) | 0.072 |
| Treatment in USD | Direct medical | | 33 (11-66) | 33 (13-69) | 31 (11-66) | 0.333 |
|  | Direct non-medical | | 252 (144-404) | 266 (169-401) | 236 (144-404) | 0.487 |
|  | Indirect costs | | 524 (167-1,059) | 583 (239-1,123) | 370 (47-1,027) | 0.206 |
| Total in USD | Direct medical | | 152 (86-267) | 163 (96-305) | 143 (74-249) | 0.222 |
|  | Direct non-medical | | 280 (156-452) | 294 (185-498) | 252 (147-414) | 0.435 |
|  | Indirect costs | | 600 (185-1,168) | 652 (265-1,308) | 478 (100-1,096) | 0.183 |
|  | Total costs | | 1,063 (644-1,823) | 1,114 (699-2,015) | 985 (621-1,516) | 0.164 |
| **Catastrophic costs (CC) from TB** | | | | | | |
|  |  | | **Total** | **CCT Cohort** | **UCT Cohort** | **p-value** |
| CC with cash transfer | | % (IQR) | 28.7 (20.9-38.0) | 38.5 (26.2-52.4) | 19.6 (11.1-32.3) | 0.031 |
| CC without cash transfer | | % (IQR) | 35.2 (26.7-44.7) | 44.2 (31.3-60.0) | 26.8 (16.7-40.0) | 0.058 |
